# Differential effects of prolonged post-fixation on immunohistochemical and histochemical staining for postmortem human brains

**DOI:** 10.3389/fnana.2024.1477973

**Published:** 2024-11-14

**Authors:** Weiya Ma, Eve-Marie Frigon, Josefina Maranzano, Yashar Zeighami, Mahsa Dadar

**Affiliations:** ^1^Cerebral Imaging Centre, Douglas Mental Health University Institute, Montreal, QC, Canada; ^2^Department of Anatomy, University of Quebec in Trois-Rivieres, Trois-Rivieres, QC, Canada; ^3^Department of Neurology and Neurosurgery, McConnell Brain Imaging Center, Montreal Neurological Institute, McGill University, Montreal, QC, Canada; ^4^Department of Psychiatry, McGill University, Montreal, QC, Canada

**Keywords:** prefrontal cortex, NeuN, GFAP, Iba1, hematoxylin, myelin, postmortem interval, brain bank

## Abstract

**Purpose:**

Immunohistochemical (IHC) and histochemical (HC) staining techniques are widely used on human brains that are post-fixed in formalin and stored in brain banks worldwide for varying durations, from months to decades. Understanding the effects of prolonged post-fixation, postmortem interval (PMI), and age on these staining procedures is important for accurately interpreting their outcomes, thereby improving the diagnosis and research of brain disorders afflicting millions of people worldwide.

**Methods:**

In this study, we conducted both IHC and HC staining on the prefrontal cortex of postmortem human brains post-fixed for 1, 5, 10, 15, and 20 years. For IHC staining, we used two antibodies for each marker: the neuron marker neuronal nuclear antigen (NeuN), the astrocyte marker glial fibrillary acidic protein (GFAP), and the microglia marker ionized calcium-binding adaptor molecule 1 (Iba1). For HC staining, we conducted hematoxylin and eosin Y (H&E), cresyl violet (CV), and Luxol fast blue (LFB) stains to examine neuropils, neurons, and myelin, respectively.

**Results:**

We observed that the intensity of NeuN, Iba1, CV, or LFB staining was negatively correlated with post-fixation durations. Conversely, we detected a positive correlation between the intensity of GFAP and H&E staining and post-fixation durations. Moreover, there was no correlation between the intensity of NeuN, GFAP, Iba1, H&E, CV, and LFB staining and PMI. Additionally, no correlation was found between these staining intensities and age, except for the intensity of GFAP immunostained by one antiserum, which was negatively correlated with age.

**Conclusion:**

Taken together, these findings suggest that prolonged post-fixation has both positive and negative effects, while age and PMI exert limited influence on these IHC and HC parameters. Therefore, it is essential to consider these differential changes when interpreting results derived from tissues with extended post-fixation durations. Furthermore, if feasible, we recommend conducting IHC and HC staining on human brains with the same post-fixation time spans and using the most optimal antibodies to mitigate the impact on subsequent analyses.

## 1 Introduction

Immunohistochemistry (IHC) and histochemistry (HC) are widely used for diagnostic and research purposes. Both approaches are essential to brain research exploring the mechanisms underlying normal brain functions as well as neurological and psychiatric disorders. Postmortem human brains are important research materials to uncover the mechanisms underlying brain-related disorders. In brain banks worldwide, it is a common practice to post-fix the donated postmortem human brains in 10% neutral-buffered formalin (NBF), i.e., 4% formaldehyde solution buffered to a neutral pH until future use. As such, most postmortem brains have usually been preserved in NBF for months, years, and decades (Vonsattel et al., 2008; Beach et al., 2015). An important concern in using these tissues is whether prolonged NBF post-fixation of postmortem brains affects the quality of subsequent IHC and HC staining. Although NBF provides excellent tissue preservation to prevent degradation in IHC (Paavilainen et al., 2010), at the same time, it leads to cross-linking of proteins and nucleic acids in tissues (Helander, 1994), thus masking antigen epitopes and reducing immunoreactivity in IHC (Arnold et al., 1996) and mRNA signals in *in situ* hybridization (Mostegl et al., 2011). Hence, prolonged NBF post-fixation is generally believed to prevent the efficient use of postmortem human brains that have been archived for years and decades. To improve IHC staining, some procedures, including enzyme-based and heat-induced antigen retrieval (HIAR), have been applied to break formalin-induced crosslinks in post-fixed tissues (Shi et al., 2011). Of these approaches, citrate buffer-based HIAR has widely been used for IHC staining on post-fixed human brains (Shi et al., 1998; Jiao et al., 1999; Ramos-Vara and Beissenherz, 2000; Leong et al., 2010). The effect of prolonged post-fixation on HIAR-IHC and/or HC staining for paraffin-embedded human brain sections was explored (Lyck et al., 2008; Webster et al., 2009; Alrafiah and Alshali, 2019; Wu et al., 2022). These studies showed that the influence of prolonged post-fixation on IHC and/or HC staining was either negative or unaltered. However, it is not clear how crosslinking affects cryosectioned tissue. Plus, the time spans of post-fixation for the examined group versus the control group from these prior studies were either very short (weeks or months) (Webster et al., 2009; Pikkarainen et al., 2010; Wu et al., 2022) or rather wide (a group with mixed post-fixation times from 1 to 20 years) (Alrafiah and Alshali, 2019). Furthermore, the detailed information on post-fixation times for the presented data was missing, and no quantitative analysis was performed (Alrafiah and Alshali, 2019), so it was not possible to systematically correlate the qualitative data with the post-fixation times.

In this study, we aimed to determine the effect of formalin post-fixation for 1, 5, 10, 15, and 20 years on IHC and HC staining on cryosections from postmortem human brains. IHC staining for neuron nuclear specific marker (NeuN), astrocyte marker glial fibrillary acidic protein (GFAP), and microglia marker ionized calcium-binding adaptor molecule 1 (Iba1) are commonly used to evaluate the three major cell types in human brain tissues. NeuN, GFAP, and Iba1 are important cell type-specific markers involved in various brain functions and in the pathogenesis of numerous brain disorders and are widely used in brain research (Reddy and Abeygunaratne, 2022). Therefore, it is important to examine whether prolonged post-fixation exerts an effect on IHC staining of the three markers in human brains. Moreover, in order to strengthen our conclusions, we applied two well-documented commercial antibodies to examine each marker in IHC studies. Hematoxylin (H) and eosin Y (E) staining, as well as cresyl violet (CV) staining, are very common HC staining methods extensively used in clinical diagnostic and brain research. The two staining techniques help to identify different types of cells and tissues in the human brain and provide important information regarding the pattern, shape, and structure of cells. Hence, it is also necessary to determine the effect of prolonged post-fixation on H&E- and CV-based HC staining in human brains.

Luxol fast blue (LFB) staining is the most common HC technique to illustrate normal myelin, assess myelin damage, and act as an indirect marker of axonal degeneration in the diagnosis and research of these diseases. Hence, it is important to evaluate the effect of prolonged post-fixation duration on LFB myelin staining in human brains.

Postmortem interval (PMI) was defined as the time elapsed after death until the samples were fixed in formalin. It is generally believed that a shorter PMI could alleviate the degradation of proteins and nucleic acids to preserve tissue and cell structures. Prior studies found that PMI over days did not seem to significantly affect IHC staining (Blair et al., 2016; Mizee et al., 2017; Krassner et al., 2023). Regarding the role of donor age in IHC staining, mixed outcomes were reported (Gonzalez-Maeso et al., 2002; Mizee et al., 2017; Krassner et al., 2023). In this study, we also examined whether PMI and donor age play a role in IHC and HC staining for human brains.

To address the above-mentioned issues, in the current study, we performed NeuN, GFAP, and Iba1 IHC staining as well as H&E, CV, and LFB HC staining in the free-floating or mounted cryosections of the prefrontal cortex (PFC) from human brains post-fixed for 1, 5, 10, 15 and 20 years. We also performed a series of linear regression analyses to examine the relationships between IHC and HC staining versus post-fixation time, PMI, and donor age.

## 2 Materials and methods

### 2.1 Postmortem human brain acquisition

Human Research Ethics Committee of Centre intégré universitaire de santé et de services sociaux (CIUSSS) de l’Ouest-de-l’Île-de-Montréal – Mental Health and Neuroscience subcommittee (2023-622). After reception of the whole brain at the Douglas-Bell Canada Brain Bank (DBCBB)^1^, postmortem human brains were collected, and hemispheres were immediately separated by a sagittal cut in the middle of the brain, brainstem, and cerebellum. One unsliced hemisphere (right or left, in alternation), 1/2 brainstem, and 1/2 cerebellum were fixed in 10% NBF (Dadar et al., 2024). For the current study, we requested PFC blocks (*n* = 20) from human brains, which have been post-fixed for 1, 5, 10, 15, and 20 years (*n* = 4 per group, in total, *n* = 20). All specimens were similarly processed and post-fixed in the same solution (i.e., 10% NBF), with the only difference in post-fixation times. DBCBB brain specimens are collected post-mortem in accordance with informed consent of the donors or the next of kin, according to tissue banking practices regulated by the Quebec Health Research Fund, and the Guidelines on Human Biobanks and Genetic Research Databases, overseen by the Douglas Research Ethics Board (Dadar et al., 2024). A blood toxicology assessment was performed, and individuals with evidence of drugs or psychotropic medications were excluded. Individuals with a known history of neurological disorders or head injuries were also excluded from this study. Demographic characteristics associated with each sample are listed in Table 1. Groups were matched as closely as possible for sex, age, race, and PMI.

**Table 1:** Characteristics of the post-mortem human brain samples used in this study.

Groups	Genders	Ages	Races	Causes of death	PMI (hours)	Post-fixation (years)
1 yr-1	M	50	Caucasian	Natural	54.50	1
1 yr-2	F	41	Caucasian	Natural	51.50	1
1 yr-3	M	19	Caucasian	Suicide	46.00	1
1 yr-4	M	33	Caucasian	Accidental	70.50	1
5 yr-1	M	53	Caucasian	Accidental	49.50	5
5 yr-2	M	59	Caucasian	Suicide	47.50	5
5 yr-3	M	55	Caucasian	Accidental	51.00	5
5 yr-4	M	59	Caucasian	Natural	46.42	5
10 yr-1	M	75	Caucasian	Natural	15.88	10
10 yr-2	M	91	Caucasian	Natural	72.68	10
10 yr-3	M	80	Caucasian	Natural	42.58	10
10 yr-4	F	84	Caucasian	Natural	10.72	10
15 yr-1	F	49	Caucasian	Suicide	59.50	15
15 yr-2	M	81	Caucasian	Accidental	98.75	15
15 yr-3	M	30	Caucasian	Suicide	51.50	15
15 yr-4	M	36	African	Natural	55.25	15
20 yr-1	F	25	Caucasian	Suicide	20.00	20
20 yr-2	M	22	Caucasian	Suicide	24.00	20
20 yr-3	F	55	Caucasian	Suicide	36.00	20
20 yr-4	F	44	Caucasian	Suicide	29.50	20

Age of the individuals (years): 19–91; average: 52.05; postmortem interval (hours): 10.72–98.75; average: 46.66.

### 2.2 Cryostat sectioning of PFC samples

After reception from the DBCBB, 20 formalin-fixed PFC-containing blocks from human brains post-fixed for 1, 5, 10, 15, and 20 years were transferred to 30% sucrose in 1X phosphate-buffered saline (PBS) for cryoprotection until sectioned on cryostat. PFC blocks were embedded in an M1 embedding medium (Fisher Scientific, Saint-Laurent, Quebec, Canada) and cut into 10- and 50-μm-thick sections (Leica, Feasterville, Pennsylvania, USA). The 10-μm-thick sections were cut from the middle part of the blocks and sequentially mounted on gelatin-pre-coated slides and stored in a -80°C freezer until they were used for HC such as H&E and CV staining. The 50-μm-thick sections were sequentially collected in cryoprotectant contained in 24-well culture plates and kept at -20°C until being used for IHC and HC, such as LBF staining.

### 2.3 NeuN, GFAP, and Iba1 immunohistochemistry

On the day of free-floating immunostaining, PFC sections were removed from the freezer and washed in 1XPBS. They were then transferred into Eppendorf tubes containing an antigen retrieval buffer (10 mM sodium citrate, 0.05% Tween 20, pH 6.0). The Eppendorf tubes were heated in boiling water (100 to 110°C) in a 1-liter beaker for 20 min. Then, the tubes were cooled down on ice, and sections were rinsed with cold double-distilled (dd) H_2_O for 10 min. The sections were then placed into a 24-well culture plate containing 1X PBS with 0.05% Triton-100 (PBS + T). To eliminate endogenous peroxidase in the tissue, the sections were quenched in 0.03% H2O2 for 15 min, followed by incubation in 50% ethanol for 1 hour to enhance tissue penetration by antisera.

Subsequently, the sections were incubated for 2 hour in a blocking solution of 10% normal goat serum (NGS) and 1% bovine albumin diluted in PBS + T to block the endogenous non-specific antigenic sites. Finally, the sections were incubated in the following primary antibodies: a mouse antibody against the neuronal marker NeuN (1:5000, Abcam, Cat. ab10424, Toronto, Ontario, Canada), a rabbit antibody against another neuron marker NeuN (1:5000, Abcam, ab177487), a rabbit antiserum against the astrocyte marker GFAP (1:5000, Novus Biologicals, Cat. NB300-141, Centennial, Colorado, USA), another rabbit antiserum against GFAP (1:2000, Abcam, Ab68428), a rat antibody against the microglia marker Iba1 (1:2000, Abcam, ab283346), or a rabbit antibody against Iba1 (1:2000, Abcam, ab178847). This incubation lasted 18 h at room temperature. Details regarding the primary antisera used are listed in Table 2.

**Table 2:** Summary of primary antisera used in this study.

Name	Source	Cat #	species	Dilution
Anti-MsxNeuN	Abcam	ab104224	Mouse	1:5000
Anti-RbxNeuN	Abcam	ab177487	Rabbit	1:5000
Anti-GFAP(nb)	Novus Biological	NB300141	Rabbit	1:5000
Anti-GFAP(ab)	Abcam	ab68428	Rabbit	1:2000
Anti-RtxIba1	Abcam	ab283346	Rat	1:2500
Anti-RbxIba1	Abcam	ab178847	Rabbit	1:2000

Then, the sections were next incubated for 1 hour in either a biotinylated goat anti-mouse, biotinylated goat anti-rabbit IgG, or biotinylated goat anti-rat IgG (1:200, MJS Biolynx Inc., Brockville, Ontario, Canada). The sections were then processed using an Elite ABC kit (MJS Biolynx Inc.) for another 1 hour, according to the manufacturer’s instructions. Finally, the immunoprecipitates were developed using 3,3-diaminobenzidine (DAB, Sigma-Aldrich, Oakville, Ontario, Canada) as the chromogen, enhanced using the glucose oxidase-nickel-DAB method (Shu et al., 1988).

The DAB reaction was carried out for 3 to 10 min, depending on the antibodies used. However, the same incubation time was applied to all sections across the groups for each IHC staining. A subjective criterion was followed to determine the approximate time to stop the DAB reaction under the microscope. The sections from the 1-year group were typically used to monitor the appearance of reliable immunoreactive (IR) profiles in the relevant regions. However, sections from other groups, such as the 20-year group, were also monitored to ensure a balanced evaluation.

Once reliable IR profiles were observed, the reaction was halted for all groups. Specifically, DAB reaction times lasted for 10 min for MsxNeuN and RbxNeuN staining, 7 min for RtxIba1 staining, 5 min for RbxIba1 and GFAP(nb) staining, and 3 min for GFAP(ab) staining. Between incubations, PFC sections were thoroughly washed in PBS + T twice. Finally, sections were mounted on pre-cleaned Super Plus glass slides (Fisher Scientific) or gelatin-coated slides, dehydrated in ascending ethanol solutions, cleared in xylene, and cover-slipped with a xylene-based mounting medium (Micromount, Leica).

After air drying overnight, the sections were observed under a bright field microscope (Olympus Microscopes, 1×73, PIF, Montreal, Canada), and digital images of the immunoreactive cells were captured using CellSens software (Olympus). As a control check, omission of primary antisera, such as anti-GFAP(ab) (Supplementary Figure 1A) or biotinylated goat anti-rabbit IgG (Supplementary Figure 1B), in the tested human brain sections from the 5-year group resulted in negative immunostaining. For the control staining, all incubation times and conditions remained the same as those without the omission of the primary antisera or the linking IgG.

### 2.4 Hematoxylin and eosin Y staining and cresyl violet staining

On the day of HC staining, slides mounted with the 10-μm-thick PFC sections from the five groups were taken out from the -80°C freezer and air dried for about 30 min to completely remove the moisture.

For H&E staining, sections were directly stained with 0.1% Mayers hematoxylin solution (Sigma, Cat. MHS-16) for 10 min before bluing in warm running tape water (pH 7.8) for 5 min. Sections were stained with 0.5% ethanol eosin Y solution (Sigma, Cat. HT110116) for 30 s. Sections were then dipped in ddH_2_O, 50, and 70% ethanol briefly, then dehydrated in 95% ethanol and 100% ethanol (2x, 5 min each) and cleared in xylene (2x, 5 min each). Finally, slides were cover-slipped, aired, dried, and observed under the microscope, as mentioned above. The nuclei of stained cells appeared to be dark blue, the cytoplasm was pink, and the erythrocytes were stained bright red. HE provides a good distinction between vascular structures and neuropil.

For CV staining, sections were incubated in 100% ethanol for 5 min and in 95% ethanol for 3 min. Then, the sections were rehydrated in 70% ethanol for 30 s and in ddH_2_O for 30 s. Sections were stained in 0.5% CV solution for 4 min. Sections were rinsed in ddH_2_O briefly before being differentiated in 70% acetified ethanol for 30 s. Sections were then dehydrated in 90 and 100% ethanol (2x, 2 min each). Finally, sections were cleared in xylene (2x, 5 min each). Slides were cover-slipped, air-dried, and observed under the microscope, as mentioned above. CV-stained Nissl substances in the cytoplasm of neurons and neuroglia appeared to be purple.

### 2.5 Luxol fast blue-eosin Y- cresyl violet staining

On the day of LFB-EY-CV staining, 50 μm-thick human PFC sections in cryoprotectant were taken out of a -20°C freezer, rinsed in PBS, mounted on the gelatin-coated slides, and air dried for about 2 hour to completely remove the moisture. Sections were soaked in xylene (2x, 15 min each), 100, and 95% ethanol (2x, 5 min each) to de-fat the myelin in PFC sections. Sections were then incubated in 1% LFB solution (Luxol fast blue stain kit, Abcam, Cat. Ab150675) at 45°C overnight. On the second day, sections were briefly rinsed in 95% ethanol and ddH_2_O (5 s each), differentiated in 0.05% lithium carbonate solution, 70% (2x, 30 s each), and rinsed in ddH_2_O briefly. The differentiating procedures were repeated until a sharp contrast between the dark blue-white matter and the colorless gray matter was observed. This criterion was used in well-documented online protocols^2^. Briefly rinsed in 70% ethanol, sections were stained in 0.05% acidified ethanol EY for 30 s. After a brief wash in ddH_2_O, sections were stained in 0.5% CV solution for 1 min. Then, sections were rinsed in ddH_2_O briefly, dehydrated through 95 and 100% ethanol, and cleared in xylene (2x, 5 min each). Section-mounted slides were cover-slipped, air-dried, and observed under a microscope, as mentioned above. Myelin in the white matter of PFC sections was stained as dark blue or blue by LFB but was sparse or absent in the gray matter, particularly absent in the superficial layers. Neuropil, or the cytoplasm of cells, was stained pink, while the red blood cells in the blood vessels were stained bright red by EY. The cytoplasm of neurons was specifically stained by CV as purple.

### 2.6 Image capture, quantification, and statistical analysis for IHC- and HC-stained sections

In addition to the same staining procedures for all sections from the five groups, the setup for image capture was consistent for all PFC sections from the five groups. Bright-field images of IHC- or HC-stained profiles were captured at the same magnification (20X) with the same threshold of illumination or exposure time on the Olympus microscope (1×73 PIF). Each captured field results in an image with a dimension of 650 μm x 475 μm. Five fields were randomly selected from Lamina II (external granule cell layer) to Lamina III (external pyramidal cell layer) of 2 to 3 stained PFC gray matter sections of each sample from 5 groups. LII and LIII basically represented the overall expression levels in the PFC gray matter. For LFB staining, five fields were selected only in the deep white matter enriched with myelin. Large blood vessels and empty spaces were avoided on purpose.

All quantification was performed by using ImageJ software (ImageJ ver.1.53e, Wayne Rasband and contributors, National Institutes of Health, USA)^3^. All images were measured in black and white (B&W) mode (8-bits). The values of average optical intensity measured fell into the 1 to 255 range of the grayscale. For each B&W image, the threshold (set up by default histogram calculation) was applied to mask background staining and empty space, if any. Thus, only the mean average optical intensity of defined IHC or HC-stained profiles was measured for each image.

The intensity measurements from five images were averaged for each brain sample. The mean intensities from all groups (1, 5, 10, 15, and 20 years of post-fixation) were statistically compared using one-way ANOVA, followed by Student–Newman–Keuls multiple comparison tests, implemented using SigmaPlot V15 (Grafiti, St. Palo Alto, California, USA). Linear regression analysis was conducted to examine the relationships between the intensity of each staining and variables such as post-fixation years, donor age, and PMI for all samples across the five groups (*n* = 20), also using SigmaPlot v15 (Grafiti). The software automatically calculated *p*-and *R*-values. A significance level of *p* < 0.05 was established for all analyses. Detailed measurement data and statistical analysis can be found in the Supplementary materials and Methods section.

## 3 Results

### 3.1 Effect of prolonged post-fixation on NeuN, GFAP, and Iba1 IHC staining

**Figure 1:**
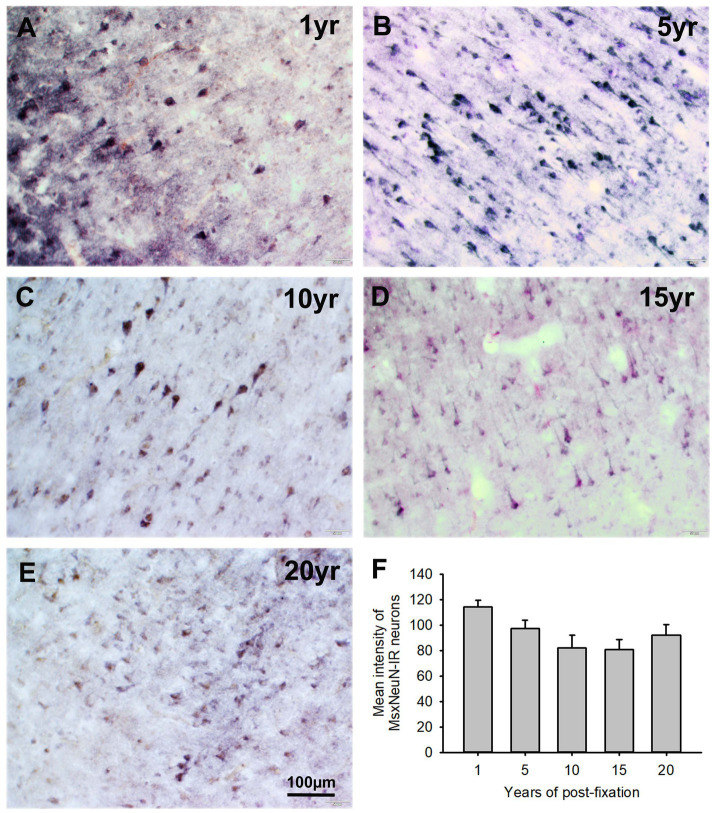
Expression of MsxNeuN-IR neurons in the gray matter of the 50-μm-thick PFC sections of human brains post-fixed for 1, 5, 10, 15 and 20 years. Numerous MsxNeuN-IR neuronal profiles were distributed throughout all layers in the gray matter of PFC sections of human brains post-fixed from 1 to 20 years **(A–E)**. Immunoprecipitates were localized not only in the nuclei but also in the cytoplasm of stained neurons. Quantitatively, a trend of reduction was noticed, but the mean MsxNeuN-IR intensity among the five groups was not significantly different **(F)**. Mean ± SEM, *n* = 4, one-way ANOVA with Student–Newman–Keuls multiple comparison test. The significance level was set at *p* < 0.05.

**Figure 2:**
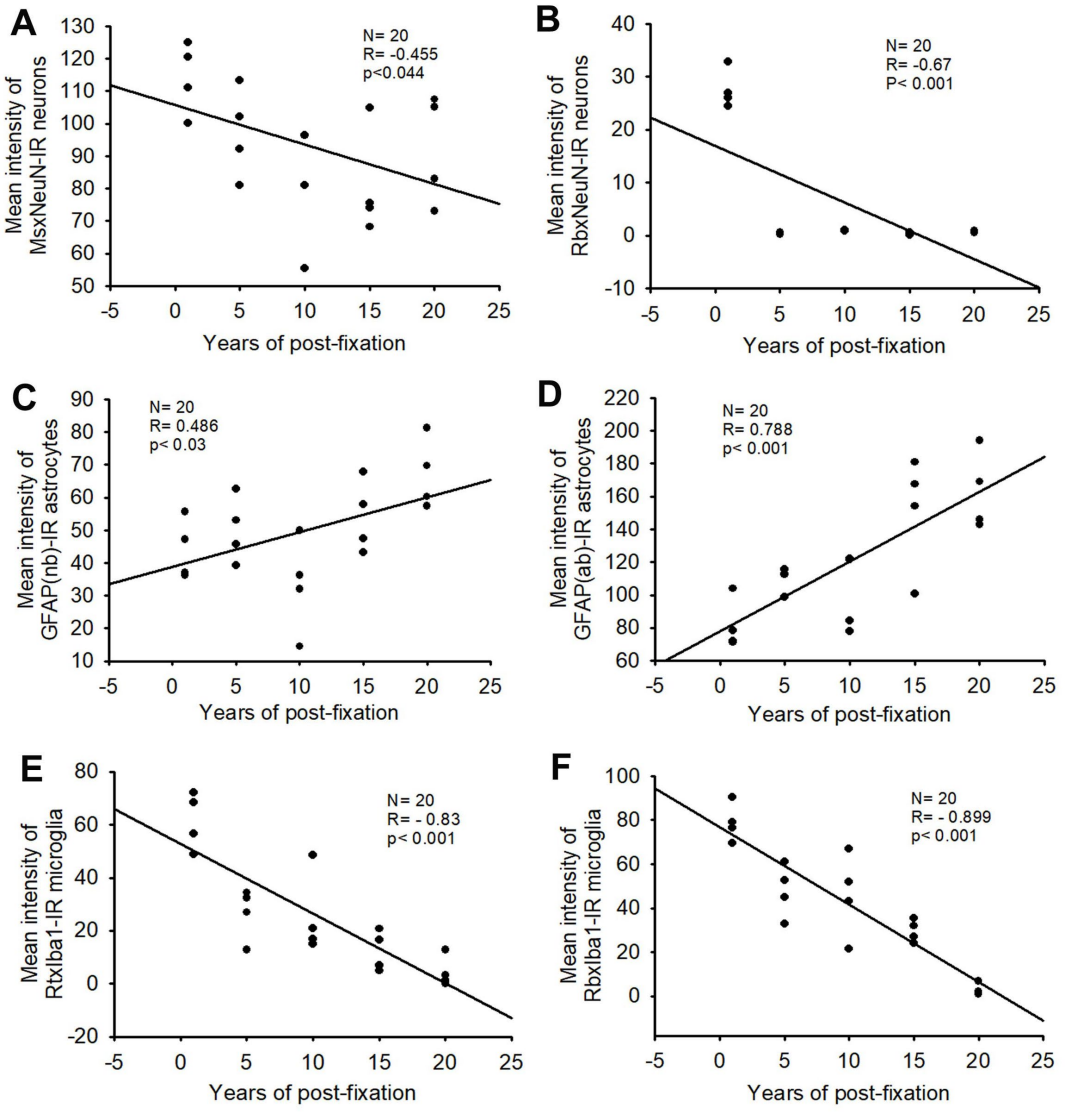
Linear regression analysis of the correlation between the mean intensity of MsxNeuN-IR neurons **(A)**, RbxNeuN-IR neurons **(B)**, GFAP(nb)-IR **(C)** and GFAP(ab)-IR astrocytes **(D)**, RtxIba1-IR **(E)** and RbxIba1-IR microglia **(F)** in PFC of human brains verse the post-fixation years. A significant negative correlation was found between the mean intensities of MsxNeuN-IR neurons (**A**, *p* < 0.05, *R* = −0.455), RbxNeuN-IR neurons (**B**, *p* < 0.001, *R* = −0.67), RtxIba1-IR microglia (**E**, *p* < 0.001, *R* = −0.83) or RbxIba1-IR microglia (**F**, *p* < 0.001, *R* = −0.899) versus the post-fixation years. However, a significant positive correlation was detected between the mean intensities of GFAP(nb)-IR (**C**, *p* < 0.03, *R* = 0.486) or GFAP(ab)-IR astrocytes (**D**, *p* < 0.001, *R* = 0.788) versus the post-fixation years. Linear regression analysis, *n* = 20. The significance level was set as *p* < 0.05.

In the current study, we used two monoclonal antibodies raised against NeuN provided by Abcam (see Table 2). Using the mouse monoclonal anti-NeuN antibody (Msx) for the 50-μm-thick PFC section from the five post-fixation groups, we observed that abundant MsxNeuN-immunoreactive (IR) neurons were present in the gray matter of human brains post-fixed from 1 to 20 years (Figures 1A–E). MsxNeuN-IR neurons exhibited typical and clear neuron morphology, with IR mainly localized in the nuclei and also seen in the cytoplasm. On quantitative analysis, the intensities of MsxNeuN-IR neurons among the five groups were not significantly different despite a trend of reduction (Figure 1F). However, linear regression analysis showed a significant negative correlation between the intensity of MsxNeuN-IR neurons and the post-fixation years (Figure 2A, *p* < 0.05, *R* = −0.455).

**Figure 3:**
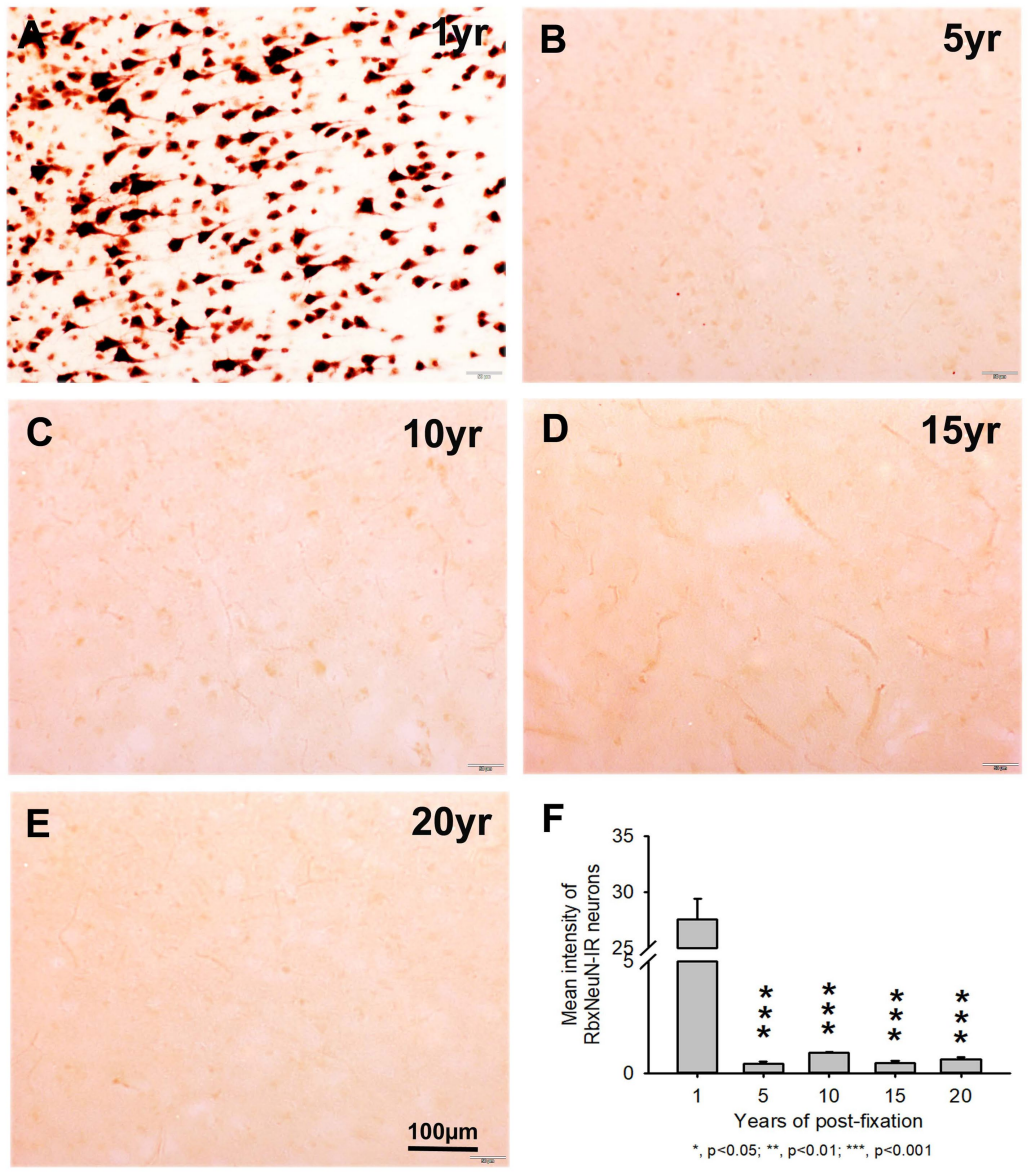
Expression of RbxNeuN-IR neurons in the gray matter of the 50-μm-thick PFC sections of human brains post-fixed for 1, 5, 10, 15, and 20 years. Abundantly and strongly stained RbxNeuN-IR neurons were only observed in the PFC of the 1-year group **(A)**. However, reliable RbxNeuN-IR neurons were almost invisible in the PFC of all other groups **(B–E)**. Quantitatively, the intensity of RbxNeuN-IR neurons from the 5, 10, 15, and 20-year groups was very significantly lower than from the 1-year group (**F**, *p* < 0.001). Mean ± SEM, *n* = 4, one-way ANOVA with Student–Newman–Keuls multiple comparison test. The significance level was set at a *p*-value of <0.05.

We also used another rabbit monoclonal anti-NeuN antiserum (Rbx) from Abcam (Table 2) to examine the 50-μm-thick PFC section of all groups. This was the same antibody used in a prior study that showed a negative correlation between NeuN-IR and post-fixation time (Wu et al., 2022). Abundant RbxNeuN-IR neurons were only found in the gray matter of PFC sections from the 1-year group (Figure 3A). Reliable RbxNeuN-IR neurons were invisible in the 5, 10, 15, and 20-year groups (Figures 3B–E). Quantitatively, the intensity of RbxNeuN-IR neurons was significantly lower in the PFC of the 5, 10, 15, and 20-year groups than in the 1-year group (Figure 3F, *p* < 0.001). Linear regression analysis showed a significant negative correlation between the intensity of RbxNeuN-IR neurons and the post-fixation years (Figure 2B, *p* < 0.001, *R* = -0.67). Together, the above observations suggest that prolonged post-fixation reduces NeuN IHC staining in human brains.

**Figure 4:**
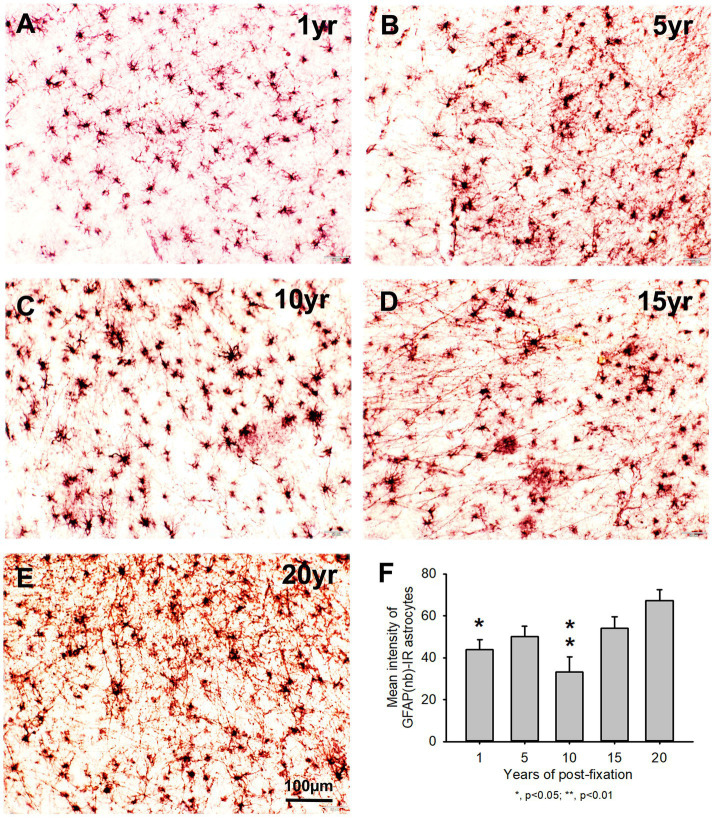
Distribution of GFAP(nb)-IR astrocytes in the 50-μm-thick PFC sections of human brains post-fixed for 1, 5, 10, 15 and 20 years. GFAP(nb)-IR astrocytes were abundantly distributed throughout the gray and white matter of PFC from human brains post-fixed for 1 to 20 years **(A–E)**. GFAP(nb)-IR astrocytes exhibited typical characteristics of astrocytes, such as a stellate soma with rather long and branched processes. Quantitative analysis revealed that the mean intensity of GFAP(nb)-IR astrocytes from the 1 and 10-year groups was significantly lower than from the 20-year group (**F**, *p* < 0.05, and *p* < 0.001, respectively). No significant difference was detected among the 5, 15 and 20-year groups **(F)**. Mean ± SEM, *n* = 4, one-way ANOVA with Student–Newman–Keuls multiple comparison test. The significance level was set at a *p*-value of <0.05.

**Figure 5:**
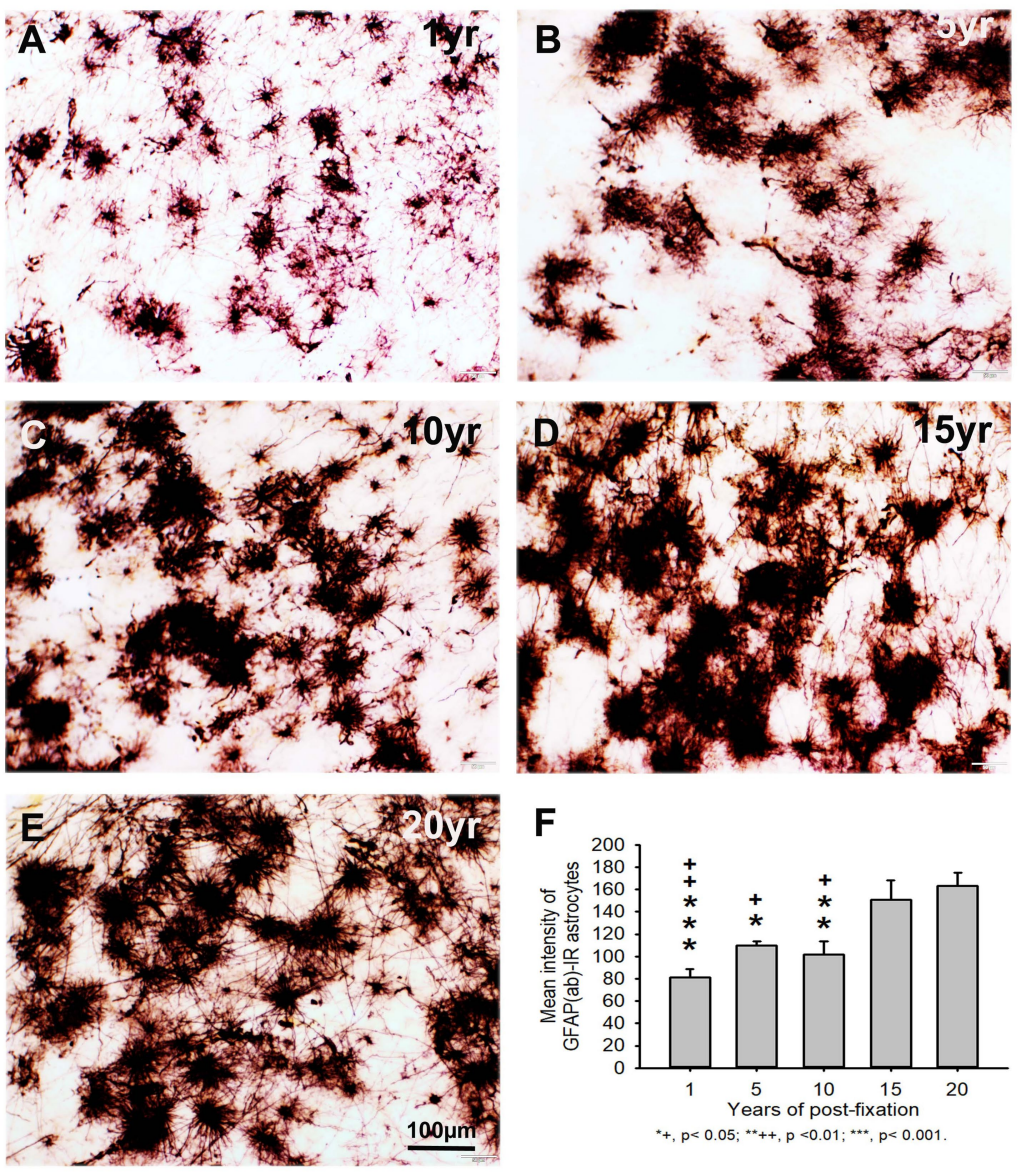
Distribution of GFAP(ab)-IR astrocytes in the 50-μm-thick PFC sections of human brains post-fixed for 1, 5, 10, 15 and 20 years. GFAP(ab)-IR astrocytes were abundantly distributed throughout the gray and white matter of PFC from human brains post-fixed for 1, 5, 10, 15, and 20 years **(A–E)**. GFAP(ab)-IR astrocytes also exhibited typical characteristics of astrocytes, such as a stellate soma with rather long and branched processes. Much stronger GFAP(ab)-IR astrocytes were observed in all groups, particularly in the groups of prolonged post-fixation **(B–E)**. Quantitative analysis revealed that the intensity of GFAP-IR astrocytes from the 1, 5, and 10-year groups was significantly lower than from the 15-year group (**F**, +*p* < 0.05 to ++*p* < 0.01) and from the 20-year group (**F**, *p < 0.05 to *p* < 0.001). No significant difference was detected among the 1, 5, 10, and 15-year groups **(F)**. Mean ± SEM, *n* = 4, one-way ANOVA with Student–Newman–Keuls multiple comparison test. The significance level was set at *p* < 0.05.

Using two rabbit anti-GFAP antibodies from Novus Biological (GFAP(nb)) and from Abcam (GFAP(ab)), we found that GFAP(nb)- or GFAP(ab)-IR astrocytes were abundantly distributed throughout the gray and white matter of PFC sections from human brains post-fixed for 1, 5, 10, 15 and 20 years (Figures 4A–E, 5A–E). Both GFAP(nb)-IR and GFAP(ab)-IR profiles exhibited typical characteristics of astrocytes, i.e., stellate soma with numerous rather long processes and branches. Much stronger GFAP(ab)-IR astrocytes were observed in all groups, particularly in the groups of prolonged post-fixation (Figures 5B–E). Quantitatively, the intensity of GFAP(nb)-IR astrocytes from the 1 and 10-year groups was significantly lower than in the 20-year group (Figure 4F, *p* < 0.05 and *p* < 0.001, respectively). No significant difference in this value was detected among the 5, 15, and 20-year groups (Figure 4F). Quantitative analysis revealed that the intensity of GFAP(ab)-IR astrocytes from the 1, 5, and 10-year groups was significantly lower than from the 15-year group (Figure 5F, *p* < 0.05 to *p* < 0.01) and from the 20-year group (Figure 5F, *p* < 0.05 to *p* < 0.001). No significant difference was detected among the 1, 5, 10, and 15-year groups (Figure 5F). Linear regression analysis revealed a significant positive correlation between the intensity of GFAP(nb) (Figure 2C, *p* < 0.03, *R* = 0.486) or GFAP(ab) (Figure 2D, *p* < 0.001, *R* = 0.788) -IR astrocytes and the post-fixation times. Together, these data suggest that prolonged post-fixation enhances GFAP IHC staining for human brains.

**Figure 6:**
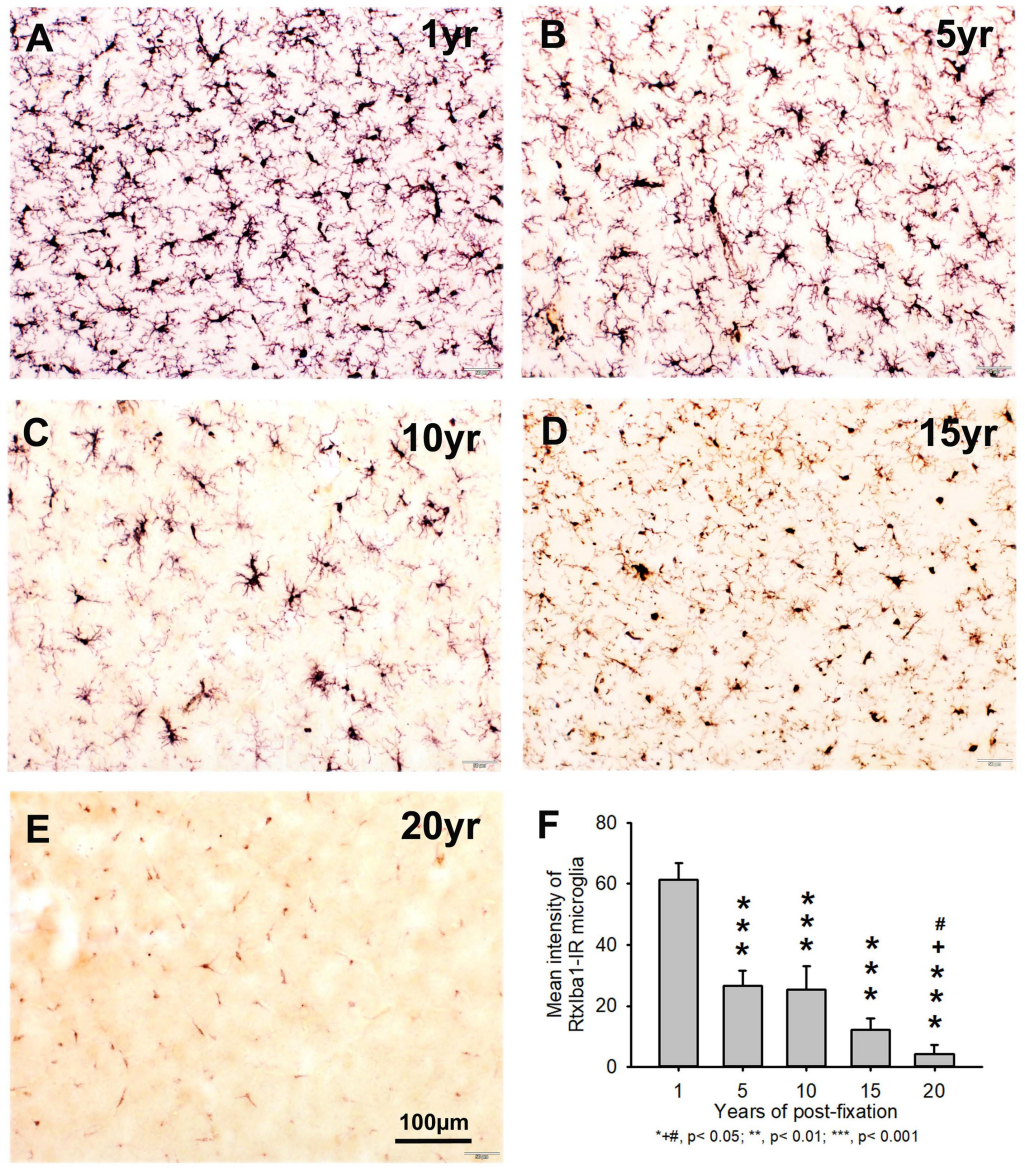
Distribution of RtxIba1-IR microglia in the 50-μm-thick PFC sections of human brains post-fixed for 1, 5, 10, 15 and 20 years. RtxIba1-IR microglia were distributed throughout the gray and white matter of PFC of the human brain post-fixed for 1 **(A)**, 5 **(B)**, 10 **(C)**, 15 **(D)**, and 20 **(E)** years. Quantitatively, the mean intensities of RtxIba1-IR microglia from the 5-, 10-, 15-, and 20-year groups were very significantly reduced compared to the 1-year group (**F**, ***p < 0.001). Moreover, the mean intensity of RtxIba1-IR microglia from the 20-year group was significantly lower than from the 5- and 10-year groups (**F**, +# *p* < 0.05). No significant difference was detected among the 5-, 10-, and 15-year groups. Mean ± SEM, *n* = 4, one-way ANOVA with Student–Newman–Keuls multiple comparison test. The significance level was set at *p* < 0.05.

**Figure 7:**
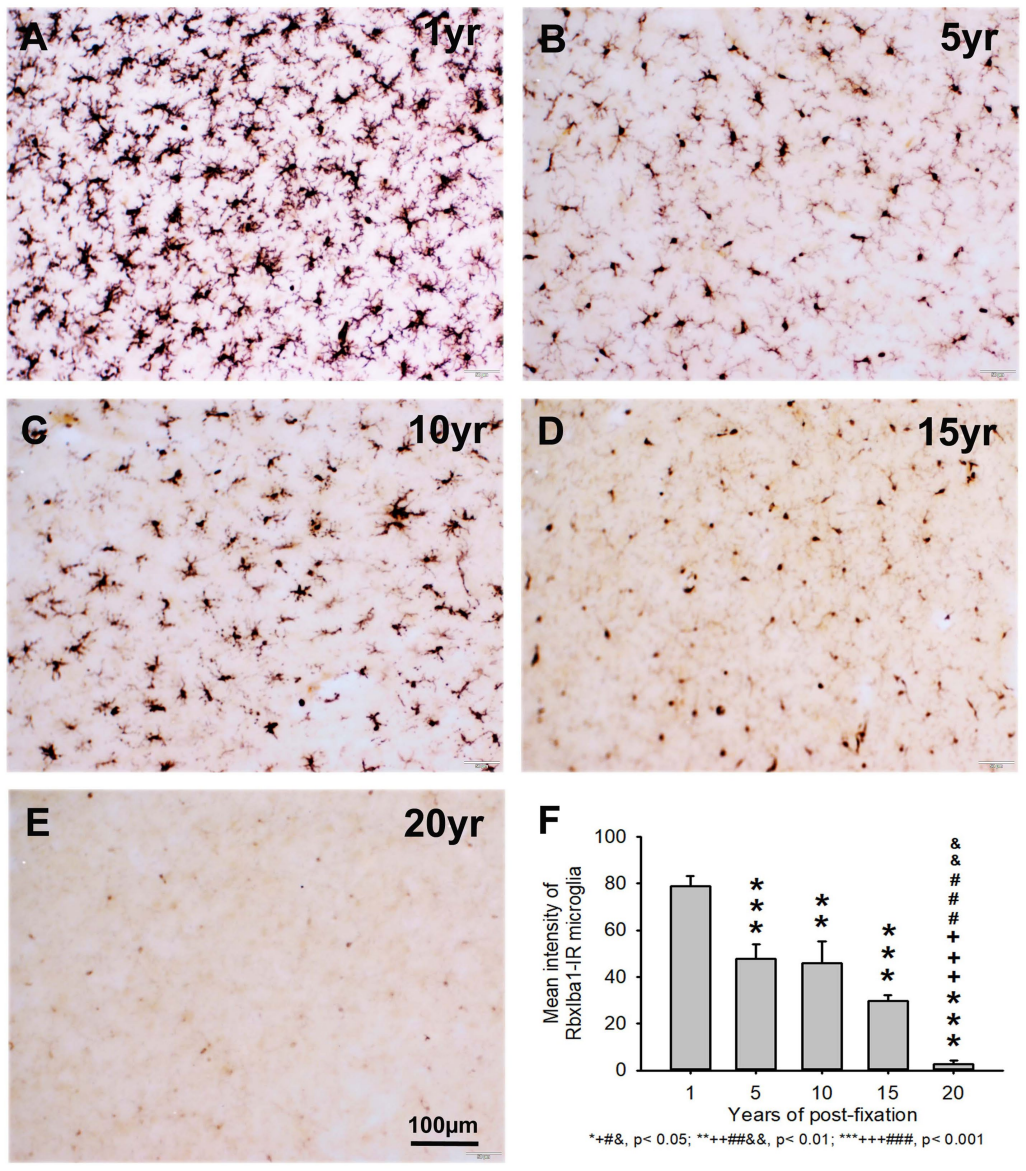
Distribution of RbxIba1-IR microglia in the 50-μm-thick PFC sections of human brains post-fixed for 1, 5, 10, 15 and 20 years. RbxIba1-IR microglia were distributed throughout the gray and white matter of PFC of human brains post-fixed for 1 **(A)**, 5 **(B)**, 10 **(C)**, 15 **(D)**, and 20 **(E)** years. Quantitatively, the mean intensity of RbxIba1-IR microglia from the 5-, 10-, 15-, and 20-year groups was very significantly reduced compared to the 1-year group (**F**, ***p < 0.001). Moreover, the mean intensity of RbxIba1-IR microglia from the 20-year group was significantly lower than from the 5- (**F**, +++*p* < 0.001), 10- (**F**, ###*p* < 0.001) and 20- (**F**, &&*p* < 0.01)-year groups. No significant difference was detected among the 5-, 10- and 15-year groups. Mean ± SEM, *n* = 4, one-way ANOVA with Student–Newman–Keuls multiple comparison test. The significance level was set at *p* < 0.05.

In the current study, we also used two anti-Iba1 monoclonal antibodies from Abcam, with one raised in rat (RtxIba1) and another one raised in rabbit (RbxIba1) (Table 2), for IHC staining on the 50-μm-thick PFC sections of human brains post-fixed for 1, 5, 10, 15 and 20 years. We found that both RtxIba1-IR and RbxIba1-IR microglia were observed throughout the gray and white matter of the PFC of the human brain post-fixed for 1 to 20 years (Figures 6A–E, 7A–E). Both RtxIba1-IR and RbxIba1-IR microglia exhibited typical characteristics of microglia, with small irregular soma and multiple short processes. Quantitatively, the intensity of RtxIba1-IR microglia from the 5-, 10-, 15-, and 20-year groups was very significantly reduced compared to the 1-year group (Figure 6F, *p* < 0.001). Moreover, the intensity of RtxIba1-IR microglia from the 20-year group was significantly lower than from the 5- and 10-year groups (Figure 6F, *p* < 0.05). No significant difference was detected among the 5-, 10-, and 15-year groups.

Similarly, the intensity of RbxIba1-IR microglia from the 5-, 10-, 15- and 20-year groups was very significantly reduced compared to the 1-year group (Figure 7F, *p* < 0.001). Moreover, the intensity of RbxIba1-IR microglia from the 20-year group was significantly lower than in the 5- (Figure 7F, *p* < 0.001), 10- (Figure 7F, *p* < 0.001), and 20- (Figure 7F, *p* < 0.01) year groups. No significant difference was detected among the 5-, 10- and 15-year groups (Figure 7F). Linear regression analysis revealed a significant negative correlation between the intensity of RtxIba1-IR microglia (Figure 2E, *p* < 0.001, *R* = −0.83) or RbxIba1-IR microglia (Figure 2F, *p* < 0.001, *R* = −0.899) versus the post-fixation years. Together, these observations suggest that prolonged post-fixation reduces Iba1 IHC staining in human brains.

### 3.2 Effect of prolonged post-fixation on hematoxylin and eosin Y staining

**Figure 8:**
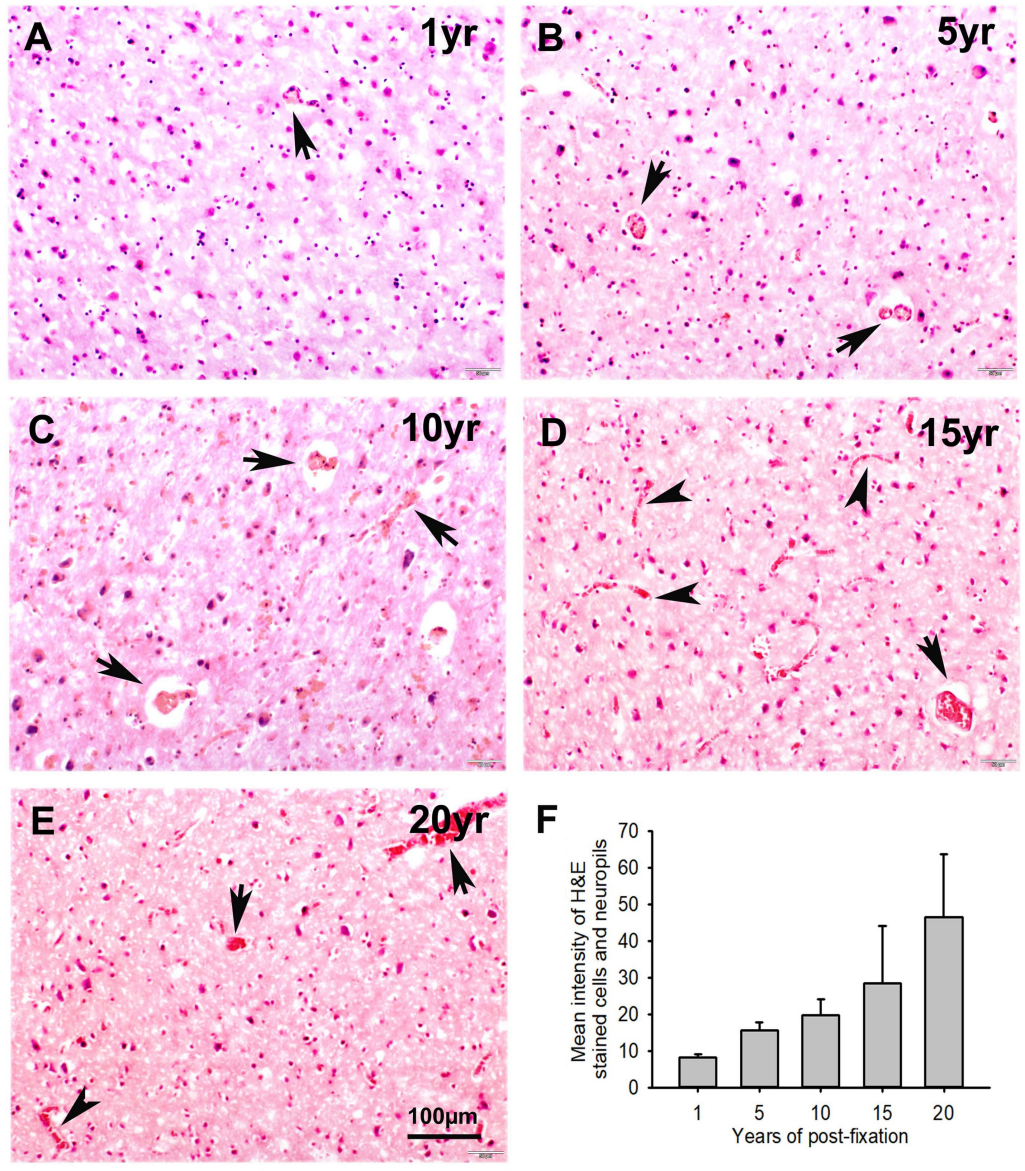
Hematoxylin (H) and Eosin Y (E) staining for the 10-μm-thick PFC sections of human brains post-fixed for 1, 5, 10, 15, and 20 years. In H&E-stained PFC sections **(A–E)**, the nuclei of cells were stained as dark blue by H, while the cytoplasm was stained as pink by E. In contrast, the red blood cells in the small vessels (**A**–**E**, arrows) and capillaries (**D**,**E**, arrowheads) were stained by E as bright red, which is easy to identify in the neuropils. Quantitatively, although a trend of reduction was evidently noticed, the mean intensities of H&E staining among the five groups were not significantly different **(F)**. Mean ± SEM, *n* = 4, one-way ANOVA with Student–Newman–Keuls multiple comparison test. The significance level was set at *p* < 0.05.

**Figure 9:**
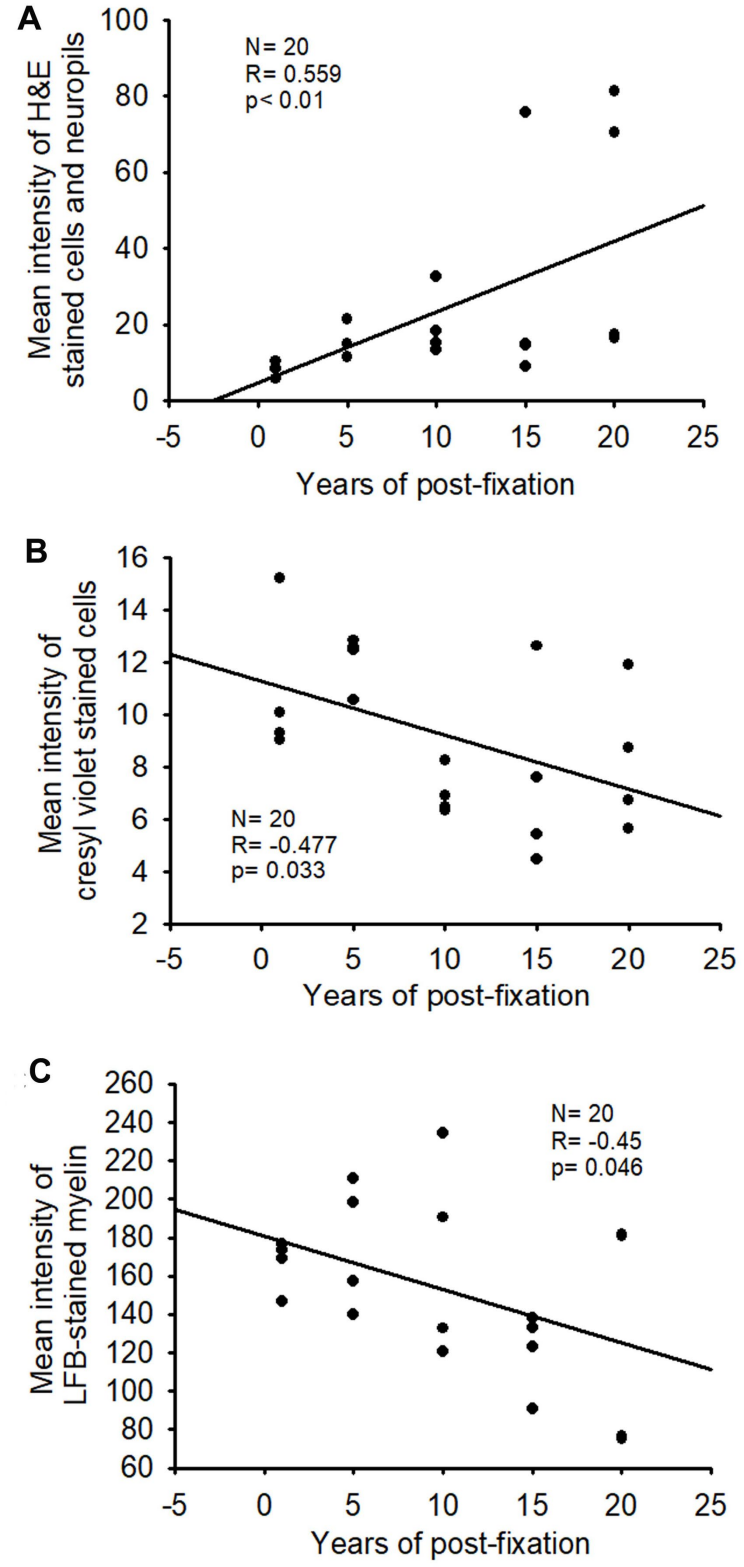
Linear regression analysis of the correlation between the intensity of H&E staining **(A)**, CV staining **(B)**, or LFB staining **(C)** in PFC of human brains and the post-fixation years. A significant positive correlation was detected between the mean intensity of H&E staining and the post-fixation years (**A**, *p* < 0.01, *R* = 0.559). A significant negative correlation was observed between the mean intensity of CV staining (**B**, *p* < 0.05, *R* = −0.477) or LFB staining (**C**, *p* < 0.05, *R* = −0.45) and the post-fixation years. Linear regression analysis, *n* = 20. The significance level was set as *p* < 0.05.

In this study, we examined H&E staining for the 10-μm-thick PFC sections of human brains post-fixed for 1, 5, 10, 15, and 20 years. The nuclei of H&E-stained cells were dark blue, while the cytoplasm was pink (Figures 8A–E). In contrast, red blood cells in the small vessels (Figures 8A–E, arrows) and capillaries (Figures 8D,E, arrowheads) were stained as bright red in the neuropils. Quantitatively, although a trend of reduction in H&E staining intensity versus the post-fixation years was evidently noticed, the intensities of H&E staining among the five groups were not significantly different on statistical analysis (Figure 8F). Linear regression analysis showed a significant positive correlation between the intensity of H&E staining and the post-fixation years (Figure 9A, *p* < 0.01, *R* = 0.559). These data suggest that prolonged post-fixation enhances H&E staining for human brains.

### 3.3 Effect of prolonged post-fixation on cresyl violet staining

**Figure 10:**
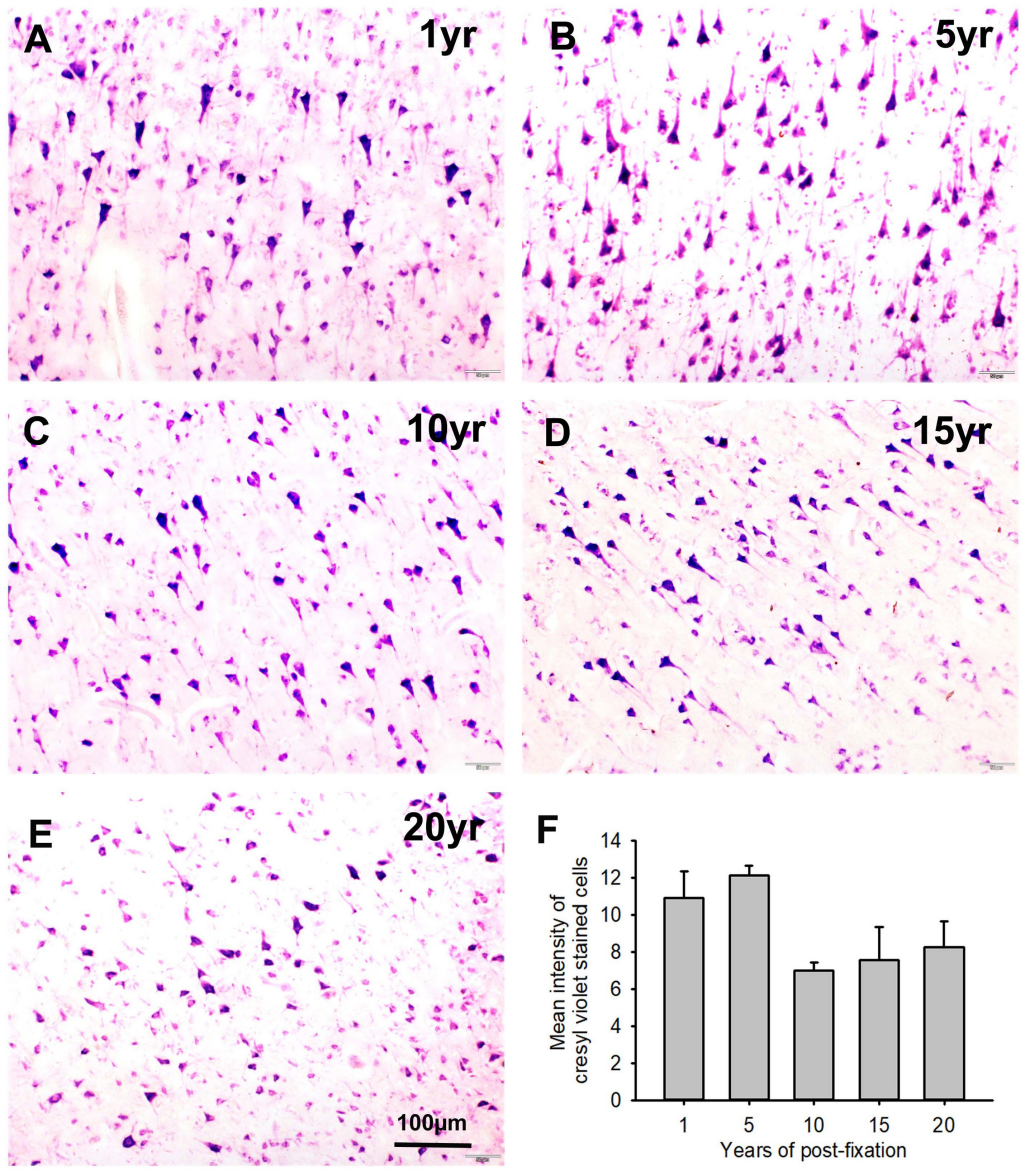
Cresyl violet staining of the 10 μm-thick PFC sections of the human brain post-fixed for 1, 5, 10, 15, and 20 years. In CV-stained PFC sections **(A–E)**, the cytoplasm of neurons was stained purple. Quantitatively, although a trend of intensity reduction was noticed, the mean intensities of CV staining among the five groups were not significantly different **(F)**. Mean ± SEM, *n* = 4, one-way ANOVA with Student–Newman–Keuls multiple comparison test. The significance level was set at *p* < 0.05.

We performed CV staining for the 10-μm-thick PFC sections from human brains post-fixed for 1, 5, 10, 15, and 20 years. CV-stained neurons were observed in the gray matter of PFC sections from all groups (Figures 10A–E). Nissl substances in the cytoplasm of neurons were stained as purple or dark purple. The abundance and intensity of CV-stained neurons were detected among the 1- and 5-year groups (Figures 10A,B). However, the abundance and intensity of CV-stained neurons in PFC of the 10-, 15-, and 20-year groups were evidently reduced (Figures 10C–E). Quantitatively, although a trend of intensity reduction was noticed, the mean intensities of CV staining among the five groups were not significantly different (Figure 10F). Linear regression analysis revealed a very significant negative correlation between the intensity of CV staining and the post-fixation years (Figure 9B, *p* < 0.05, *R* = −0.477). These data suggest that prolonged post-fixation reduces CV staining in human brains.

### 3.4 Effect of prolonged post-fixation on myelin staining

**Figure 11:**
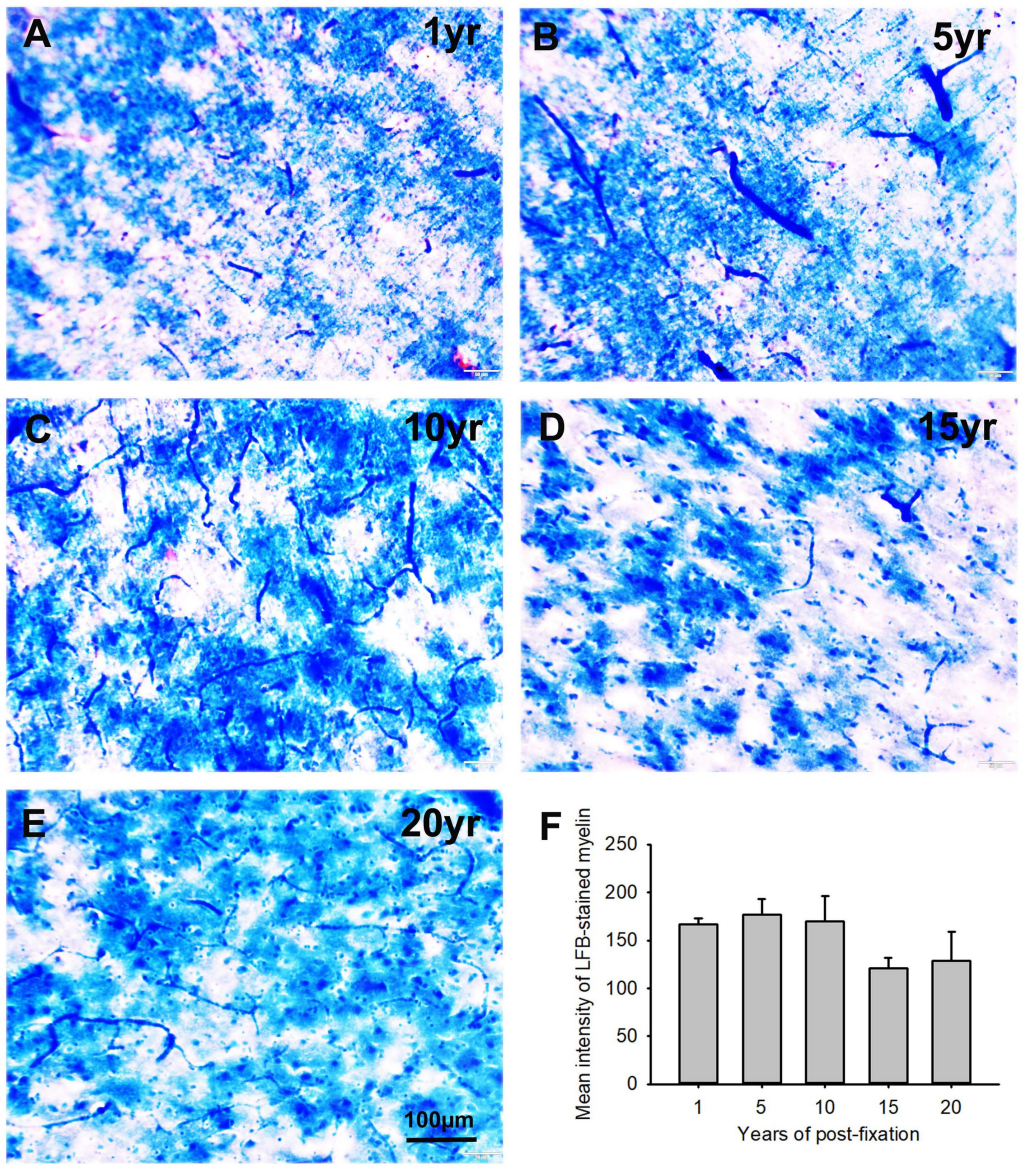
Luxol fast blue (LFB)-Eosin Y(EY)-cresyl violet (CV) staining of the 50 μm-thick PFC sections from human brain post-fixed for 1, 5, 10, 15, and 20 years. In the myelin-enriched white matter of PFC **(A–E)**, heavily LFB-stained myelin profiles were observed. The neuropil and blood vessels were counterstained by EY as pink and bright red, respectively **(A,C)**. Neurons in the myelin lacking gray matter were stained purple (not shown here). Quantitatively, although a trend of intensity reduction was noticed, the difference in the mean myelin intensities from the five groups was not statistically significant **(F)**. Mean ± SEM, *n* = 4, one-way ANOVA with Student–Newman–Keuls multiple comparison test. The significance level was set at a *p*-value of <0.05.

In this study, using the LFB-EY-CV staining protocol, we performed myelin HC staining for the 50-μm-thick PFC sections from human brains post-fixed for 1, 5, 10, 15, and 20 years. We observed that the myelin-enriched white matter of PFC from all brains of the five groups was stained as dark blue or blue by LFB, while the neuropils and blood vessels were stained as pink and red by EY (Figures 11A–E). In the gray matter (data not shown), where myelin was absent or sparse, neurons were stained by CV as purple. Quantitatively, although a trend of intensity reduction was noticed, the differences in the myelin intensities from the five groups were not statistically significant (Figure 11F). However, linear regression analysis revealed a significant negative correlation between the myelin intensity and the post-fixation years (Figure 9C, *p* < 0.05, *R* = −0.45). These data suggest that prolonged post-fixation reduces myelin staining in human brains.

### 3.5 Effects of donor age and postmortem interval on IHC and HC staining

In the current study, to examine the possible effect of donor age and PMI of brain samples on IHC and HC staining, we performed linear regression analysis (Tables 3, 4). There was a significant negative correlation between the donor age and the intensity of GFAP(nb)-IR astrocytes in the PFC of human brains (Table 3, *R* = −0.624, *p* < 0.003). However, there was no significant correlation between the donor age and the intensity of any other IHC, including GFAP(ab) or HC staining (Table 3). Moreover, we did not observe any significant correlation between PMI and the intensity of any IHC and HC staining (Table 4). These data suggest that donor age and PMI exert a limited effect on IHC and HC staining.

**Table 3:** Summary of linear regression analysis between ages and intensity of IHC and HC.

Intensity	Age	Size	*R*	*p*
MsxNeuN-IR	19–91	20	0.93	0.086
RbxNeuN-IR	19–91	20	0.357	0.122
GFAP(nb)-IR	19–91	20	-0.624	0.003
GFAP(ab)-IR	19–91	20	0.157	0.508
RtxIba1-IR	19–91	20	0.056	0.816
RbxIba1-IR	19–91	20	0.037	0.876
H&E staining	19–91	20	0.265	0.260
CV staining	19–91	20	0.356	0.124
LFB staining	19–91	20	0.142	0.551

A significant negative correlation was detected between GFAP-IR intensity and age (*R* = -0.624, *p* < 0.003). There was no significant correlation between the intensity of any other IHC and HC staining versus age.

**Table 4:** Summary of linear regression analysis between PMI and intensity of IHC and HC.

Intensity	PMI (hours)	Size	*R*	*p*
MsxNeuN-IR	10.72–98.75	20	0.008	0.975
RbxNeuN-IR	10.72–98.75	20	0.218	0.356
GFAP(nb)-IR	10.72–98.75	20	0.159	0.502
GFAP(ab)-IR	10.72–98.75	20	0.0142	0.953
RtxIba1-IR	10.72–98.75	20	0.153	0.520
RbxIba1-IR	10.72–98.75	20	0.199	0.399
H&E staining	10.72–98.75	20	0.298	0.201
CV staining	10.72–98.75	20	0.046	0.847
LFB staining	10.72–98.75	20	0.051	0.83

There was no significant correlation between the intensity of any IHC and HC staining versus PMI.

## 4 Discussion

### 4.1 Summary of major findings

In the current study, we examined NeuN, GFAP, and Iba1 IHC staining as well as H&E, CV, and LFB HC staining on PFC sections from human brains post-fixed for 1, 5, 10, 1,5, and 20 years. A negative correlation was detected between the IHC staining of NeuN and Iba1 and the HC staining of CV and LFB versus the post-fixation years. By contrast, a positive correlation was found between GFAP IHC staining and H&E HC staining versus the post-fixation times. These data suggest that prolonged post-fixation exerts differential facilitating and inhibiting effects on IHC and HC staining. Furthermore, except for a negative correlation between the donor age and GFAP(nb) IHC staining, no significant correlation was detected between donor age or PMI versus any other IHC and HC staining mentioned above. These data suggest that unlike post-fixation durations, donor age and PMI play limited roles in IHC and HC staining intensity for postmortem human brains.

### 4.2 Effects of prolonged post-fixation on NeuN, GFAP, and Iba1 IHC staining for human brains

Prior studies have demonstrated a negative correlation between NeuN-IR intensity and post-fixation times, with findings ranging from immediate post-fixation to durations of up to 3 years (Wu et al., 2022) or from 24 h to 4 months and 10 years (Lyck et al., 2008). Another prior IHC study on post-fixed swine brains showed that NeuN expression was reduced in swine brains post-fixed for 2 months, ultimately disappearing completely after 3 months post-fixation (Lundstrom et al., 2019). These prior findings suggest that prolonged post-fixation, even over a couple of months or years, suppresses NeuN expression in IHC staining.

In this study, we significantly extended the time span of examination for human brain tissues. Consistent with these prior studies, we found a negative correlation between NeuN-IR intensity and post-fixation time in human brains using two anti-NeuN antisera from Abcam. However, the two antisera produced different IHC staining patterns, although the trend related to post-fixation effects was similar. Reliable NeuN-IR neurons (MsxNeuN) were observed in all groups, including the 20-year group. However, the RbxNeuN-IR neurons, using the same antibody as in the study by Wu et al. (2022), were only present in the brains of the 1-year group.

Our data suggest that the effect of prolonged post-fixation might also depend on the specific antibodies used or on relevant molecular changes in the tissues. However, it is hard to determine whether the differing NeuN staining patterns resulted from variations in the epitopes or sequences targeted by the two anti-NeuN antibodies, especially since two anti-Iba1 antibodies yielded the same staining pattern despite potentially different epitopes or sequences. Therefore, a suitable NeuN antibody could reveal the target protein even in brain samples post-fixed for two decades, although the staining intensity was significantly reduced compared to samples from fewer years of post-fixation. As such, it is possible to compensate for the prolonged post-fixation reduced immunoreactivity with a more effective antibody. It is recommended that more antibodies be assessed and found to be the most optimal ones for performing IHC staining for postmortem brains, thus offsetting the negative effect of prolonged post-fixation on IHC staining.

GFAP is an important biomarker widely used in the diagnosis and research of neurological disorders, such as Alzheimer’s disease (Abdelhak et al., 2022; Kim et al., 2023). Astrocytosis could be an alternative pathway for the pathogenesis of AD (Pereira et al., 2021). To understand the mechanisms underlying neurological disorders, it is important to examine GFAP expression in archived human AD brains that have been post-fixed for years or even decades.

Previous studies have demonstrated that GFAP levels were reduced in human brains with post-fixation for durations ranging from 24 h to 4 months and even up to 10 years, as assessed by immunoarray techniques. However, GFAP-immunoreactive (GFAP-IR) astrocytes were still sufficiently visible in tissue fixed for 10 years when high-iron antigen retrieval (HIAR) was applied in immunohistochemistry (IHC) (Lyck et al., 2008). Another study showed that GFAP-IR intensity remained unaltered in human brains post-fixed for periods ranging from months up to 3 years, as determined by IHC staining (Wu et al., 2022).

For the first time, contrary to these studies, using two GFAP antibodies from different sources, we observed a positive correlation between GFAP(nb)-IR or GFAP(ab)-IR intensity versus post-fixation durations ranging over 1, 5, 10, 15, and 20 years. The staining patterns of both GFAP(nb) and GFAP(ab) were the same, although their staining intensities were different either due to differences in antigen sensitivity or different antibody dilutions. Indeed, we also observed abundant strongly stained GFAP-IR astrocytes in the PFC and hippocampus of human brains post-fixed for 25 years (*n* = 1, Supplementary Figure 2).

Our data strongly suggest that prolonged post-fixation exerts a positive or facilitating effect on GFAP IHC staining. The detection of GFAP in human brains post-fixed for >20 years opens new avenues for utilizing large quantities of donated and archived AD brains in research focused on this debilitating disease.

Elevation of microglia biomarkers is a consistent feature of AD brains (Hopperton et al., 2018), suggesting a crucial role of microglia activation in AD pathogenesis. Thus, it is important to determine the effect of post-fixation on microglia markers such as Iba1 in IHC studies. Prior studies using immunoassay showed that active microglia markers (CD45+ and CD68) in postmortem human brains were negatively correlated with post-fixation time (from 24 h to 4 months and up to 10 years) (Lyck et al., 2008). However, a subsequent study showed no alteration in Iba1 IHC staining detected in human brains post-fixed from weeks up to 3 years (Wu et al., 2022). Consistent with the study by Lyck et al. (2008), using two Iba1 antibodies raised in two different species, we found here a very significant negative correlation between RtxIba1-IR or RbxIba1-IR intensities versus post-fixation years. The staining pattern and abundance of both RtxIba1-IR and RbxIba1-IR were almost the same, further strengthening the reliability of our results. Together, these data suggest that extended post-fixation duration reduces Iba1 IHC staining in postmortem human brains.

It remains unknown why prolonged NBF post-fixation exerts differential positive and negative effects on IHC staining. For the negative effect, it was assumed that prolonged post-fixation progressively enhances the crosslinking between proteins to mask most antigen epitopes, thus leading to reduced immunoreactivity. On the contrary, NBF-induced protein crosslinking in human tissues might change protein conformation and expose some antigens. However, in our pilot study (Supplementary Figure 2), we observed that citrate buffer (pH6.0)-based HIAR, which breaks up formalin-induced crosslinks, enhanced GFAP(nb) IHC staining for brains post-fixed for 25 years. This observation indicates that NBF-induced crosslinks are unlikely to be the reason for the positive effect of prolonged post-fixation on GFAP IHC staining. Moreover, we still observed that prolonged post-fixation also exerts differential positive and negative effects on HC staining (see below), which further supports the assumption that more antigen exposures by NBF-induced crosslinks are unlikely the reason to explain the positive effect.

Prior studies suggest that molecular-dependent changes occurring in prolonged NBF-fixed brain tissues are likely associated with the effect of post-fixation on IHC (Lyck et al., 2008; Wu et al., 2022). Indeed, it was shown that the effects of post-fixation delays >12 h specifically relied on each antigen since IR cells could be artificially increased, decreased, or unaffected depending upon the targeted antigens (Lyck et al., 2008; Engel and Moore, 2011). Based on these findings, the differential positive and negative effect of prolonged post-fixation on IHC staining might be the reflection of the molecular changes due to NBF or other factors in brain tissues. Future studies are required to address this issue.

### 4.3 Effects of prolonged formalin post-fixation on H&E, CV, and LFB HC staining for postmortem human brains

H&E staining is the standard histological protocol as it produces a good overview of the tissue and the cellular components to clearly display different types of structures. In the current study, for the first time, we demonstrated a positive correlation between H&E staining intensity and post-fixation time, ranging from 1, 5, 10, 15, to 20 years. This observation suggests that post-fixation improves or enhances H&E HC staining. Although more intense staining does not necessarily represent better staining, particularly for H&E staining, it does indicate more affinity of tissues to the dyes and vigorousness of staining.

CV staining is a fundamental method to visualize the rough endoplasmic reticulum and ribosomes, the Nissl substance, along the course of dendrites or in neuronal perikaryons. Regarding the effect of prolonged post-fixation on CV staining, it was previously shown that the intensity of CV-stained neurons was negatively correlated with post-fixation time (from weeks to 3 years) (Wu et al., 2022). In the current study, we greatly expanded the time span to examine this issue and consistently found a negative correlation between CV staining intensity and post-fixation durations ranging over 1, 5, 10, 15, and 20 years.

In addition to magnetic resonance imaging (MRI) and IHC staining for *myelin* basic protein (*MBP*), Luxol fast blue (LFB) staining is the most common HC technique to examine myelin and axonal degeneration in diagnostic and research. Prior studies showed that NBF post-fixation of postmortem human brains for 43 and 64 days altered MRI indices of myelin images (Schmierer et al., 2010; Shatil et al., 2018). Using tissue immunoassay, it was reported that MBP-IR was reduced in human brains post-fixed from 4 months to 10 years (Lyck et al., 2008). Another more recent qualitative study showed that prolonged post-fixation of human brains (a group of 1 to 20 years) reduced myelin intensity on LFB staining compared to the control group (< 1 year) (Alrafiah and Alshali, 2019). In this study, we addressed this issue in a more systematic and quantitative way and found a negative correlation between myelin intensity and post-fixation time (1, 5, 10, 15, and 20 years) on regression analysis. However, using one-way ANOVA with multiple comparison tests, no significant difference in the myelin intensity was detected among the five groups, although the myelin intensity of the 15- and 20-year groups was evidently lower than those of the 1, 5, and 10-year groups. These data suggest that prolonged post-fixation reduces myelin staining, but this reduction in the first 10 years was not evident.

Taking the above-mentioned data into consideration, a question was raised as to why prolonged post-fixation exerts differential positive and negative effects on HC staining. Formalin-induced protein crosslinks seem suitable for explaining a stronger H&E staining as post-fixation prolongs. However, this presumption fails to interpret the negative effect of prolonged post-fixation on CV and LFB staining. Alternative theories, such as the degradation of certain proteins, i.e., myelin or Nissl substances, must be explored. Different reactivity of brain cells to different staining procedures may reflect the distinct property or nature of subcellular components or proteins exerted by prolonged exposure to NBF. Further studies are needed to address these issues.

### 4.4 Effects of donor age and postmortem interval on IHC and HC staining

In this study, except for a negative correlation between age and GFAP(nb) IHC staining, no significant correlation was observed between age or PMI versus any other IHC and HC staining. Our data was consistent with previous studies showing no correlation between PMI and IHC. For example, it was shown that immunostaining profiles for most proteins remained unchanged even after PMI of over 50 h (Blair et al., 2016), and cellular features appeared to remain intact for more extended PMI (Krassner et al., 2023). Even when RNA was degraded, the protein levels in postmortem human brains remained stable (Stan et al., 2006). Furthermore, we recently showed that PMI had no significant effects on histology quality (Frigon et al., 2024). All of these above-mentioned data suggest that PMI up to 3 days likely exerts no major effects on IHC and HC staining of postmortem brains. A recent review mentioned that the evidence for the interaction of age with PMI effects is mixed and may depend on the structural features considered (Krassner et al., 2023). Isolation of primary microglia from the human postmortem brain was not affected by donor age and PMI (Mizee et al., 2017). However, a negative correlation was reported between age and immunoreactivity for G-protein subunit G_αi1/2_-but not for subunit G_αi3_-, G_αo_-, and G_αs_-proteins (Gonzalez-Maeso et al., 2002).

In this study, we only detect a negative correlation between GFAP(nb) intensity versus donor age. However, no correlation was found between the intensity of any other IHC staining, including GFAP(ab), or any HC staining versus donor age. The differential effects of donor age on GFAP expressions revealed by two different antisera indicate that some epitopes of antigen proteins or peptides might be altered by aging so that they can only be detected by antisera containing the sequence of modified proteins or peptides. Thus, target protein or peptide markers would not be affected by donor age. If the epitope sequences modified by aging are not contained in the designed antisera, the target proteins will not be observed. Therefore, optimizing antibodies can help to exclude the influence of aging on IHC staining for brain samples after prolonged post-fixation. GFAP expression was known to be increased in aging human brains (Wruck and Adjaye, 2020; Chatterjee et al., 2021). Elevated GFAP expression is likely involved in the pathogenesis of neurodegenerative diseases such as Alzheimer’s disease, Parkinson’s disease, and amyotrophic lateral sclerosis (Palmer and Ousman, 2018). The negative involvement of aging in GFAP(nb) IHC staining indicates that to achieve consistent results, it is better to perform GFAP IHC staining on the postmortem brain within similar ranges of donor age and post-fixation durations.

### 4.5 Technical limitations and relevant solutions

It is worth mentioning that most prior studies exploring the effect of prolonged post-fixation on IHC and HC staining were performed on paraffin-embedded human brain sections. The current study, for the first time, examined this issue on both thin and thick cryosections of postmortem human brains. Although paraffin embedding provides better tissue preservation, shrinkage of tissues caused by extensive dehydration is a frequently encountered drawback. Moreover, the paraffin-embedding process also prevents the penetration of antibodies (Ramos-Vara et al., 2014). Cryosectioning is a fast and convenient technique to obtain large quantities of thin (10 to 20 μm thick) and thick (40 to 50 μm) sections in a short time. IHC for mounted thin sections or for free-floating thick sections makes staining more versatile and flexible. Following HIAR, the quality of IHC staining was comparable for both paraffin-embedded sections and cryosections (Shi et al., 2011). It was also shown that NBF post-fixation and subsequent cryosectioning did not affect the size and shape of ocular tissues (Tran et al., 2017). Our data support the reliability of using cryosections from human brains post-fixed for years and decades in research of neurological and psychiatric disorders.

In IHC and HC staining, variable background staining due to tissue preservation, antibodies, or dye affinities is a frequently encountered issue. Its effects on quantification should be eliminated or at least minimized in order to obtain a relatively objective outcome. In this study, we measured the average optical intensity for each IHC or HC-stained image using the threshold setup on ImageJ. The threshold was automatically determined on the software by calculating the histogram of each image. Only profiles over the threshold were measured, while those below the threshold were excluded. Background staining and empty space were well below the threshold compared to the presumed positive profiles. For H&E staining, intensely stained blood vessels and stasis/thromboses were frequently seen. Most of the time, they were well over the threshold and then measured to contribute to the variability of average optical intensity. However, the chance of such a situation would be the same since the images for all groups were randomly selected. Therefore, the eventual outcome should be a real reflection of H&E staining for each group.

The reliability of primary antibodies used in the current study could be an issue for IHC staining. However, all primary antisera were widely used in prior studies, and their reliability was well supported by a huge number of references from which they were used for various human or animal studies. Therefore, in the current study, we only performed negative control tests by omitting the primary antisera or the biotinylated linking IgG. These omission control tests resulted in negative staining, as shown in Supplementary Figure 1. Of course. It is better to perform the test of antigen pre-absorption or pre-neutralization for primary antibodies to justify their reliability. However, the relevant antigen proteins or peptides were not commercially available.

In the current study, although we did not detect any correlation between IHC and HC staining versus PMI, the effects of the cause of death on these staining might exist. The cause of death may impact tissue reactivity, as agonic deaths are known to provide tissue of a slightly worse nature than sudden deaths. Unfortunately, the detailed causes of death for these donated brain samples used in this study were not available. Future studies are required to address this issue.

### 4.6 Significance of the findings from the current study

We demonstrated here a positive effect of prolonged NBF post-fixation on GFAP IHC staining and H&E HC staining as well as a negative effect on NeuN, Iba1 IHC staining, and CV and IFB HC staining for PFC from postmortem human brains. The differential positive and negative effects could result from the molecular conformational changes associated with post-fixation times. Moreover, we found no correlation between the donor age or PMI with IHC and HC staining for most target proteins or structures, except for GFAP(nb). Thus, successful IHC and HC staining depends on post-fixation times, target molecules, available antibodies, and staining procedures. Therefore, it is recommended that IHC and HC staining be performed for postmortem human brains post-fixed at the same time windows, and to use the most optimal antibodies and staining procedures. Since prolonged post-fixation exerts both positive and negative effects on IHC and HC staining, depending on the target molecules, subsequent IHC or HC staining analyses could be performed in a more flexible time window of post-fixation. Since all IHC and HC staining was performed on thin or thick cryosections and reliable staining results were achieved, this study also supports the application of cryosectioning in morphological studies of postmortem brains.

The current study expands the time span of possible application of archived human brains post-fixed for years and decades for clinical diagnosis and brain research. Hence, the data collected from the current study could be used to model and adjust for the impact of post-fixation on some of the IHC and HC staining measures, if not all. Exploration of these long archived and post-fixed human brains will advance our understanding of the mechanisms underlying neurological and psychiatric disorders.

## Data Availability

Summary results for all experiments performed are provided in Table 1 in the Supplementary material.

## Footnotes

^1^https://douglasbrainbank.ca

^2^https://ihcworld.com/2024/01/26/luxol-fast-blue-staining-protocol-for-myelin/#google_vignette

^3^http://imagej.nih.gov/ij

## Supplementary material

SUPPLEMENTARY FIGURE 1 IHC omission staining. In **A**, primary antibodies were omitted, the 50μum-thick PFC sections from the 5-year-group were only incubated in biotinylated goat anti-rabbit IgG and Elite ABC solutions, and then were incubated in glucose oxidase-nickel-DAB solutions. In **B**, the sections were only incubated in a monoclonal rabbit anti-GFAP(ab) antiserum (Abcam, ab68428, 1:2000) while the biotinylated goat anti-rabbit IgG incubation was omitted. Next sections were processed in the following steps as mentioned above. All control sections were treated under the same conditions as those with the primary antisera and the linking IgG. However, following two omission tests, no reliable GFAP(ab)-IR astrocytes were observed in any region of PFC of all brain sections examined.

SUPPLEMENTARY FIGURE 2 Expression of GFAP(nb) immunostaining in the CA1 region of hippocampus of a human brain post-fixed for 25 years. Following the treatment of citrate buffer (pH6.0) based heat-induced antigen retrieval (HIAR), abundant strongly stained GFAP(nb)-IR astrocytes were observed in the CA1 of hippocampus **(A)**. However, without HIAR treatment, GFAP(nb)-IR astrocytes were less strong and less abundant in the same region **(B)** compared to that with HIAR treatment **(A)**.
