# Supplementary figures and images for: Differential effects of prolonged post-fixation on immunohistochemical and histochemical staining for postmortem human brains
Source: Front Neuroanat. 2024 Nov 14;18:1477973. doi: 10.3389/fnana.2024.1477973 (PMC11602276; doi:10.3389/fnana.2024.1477973)

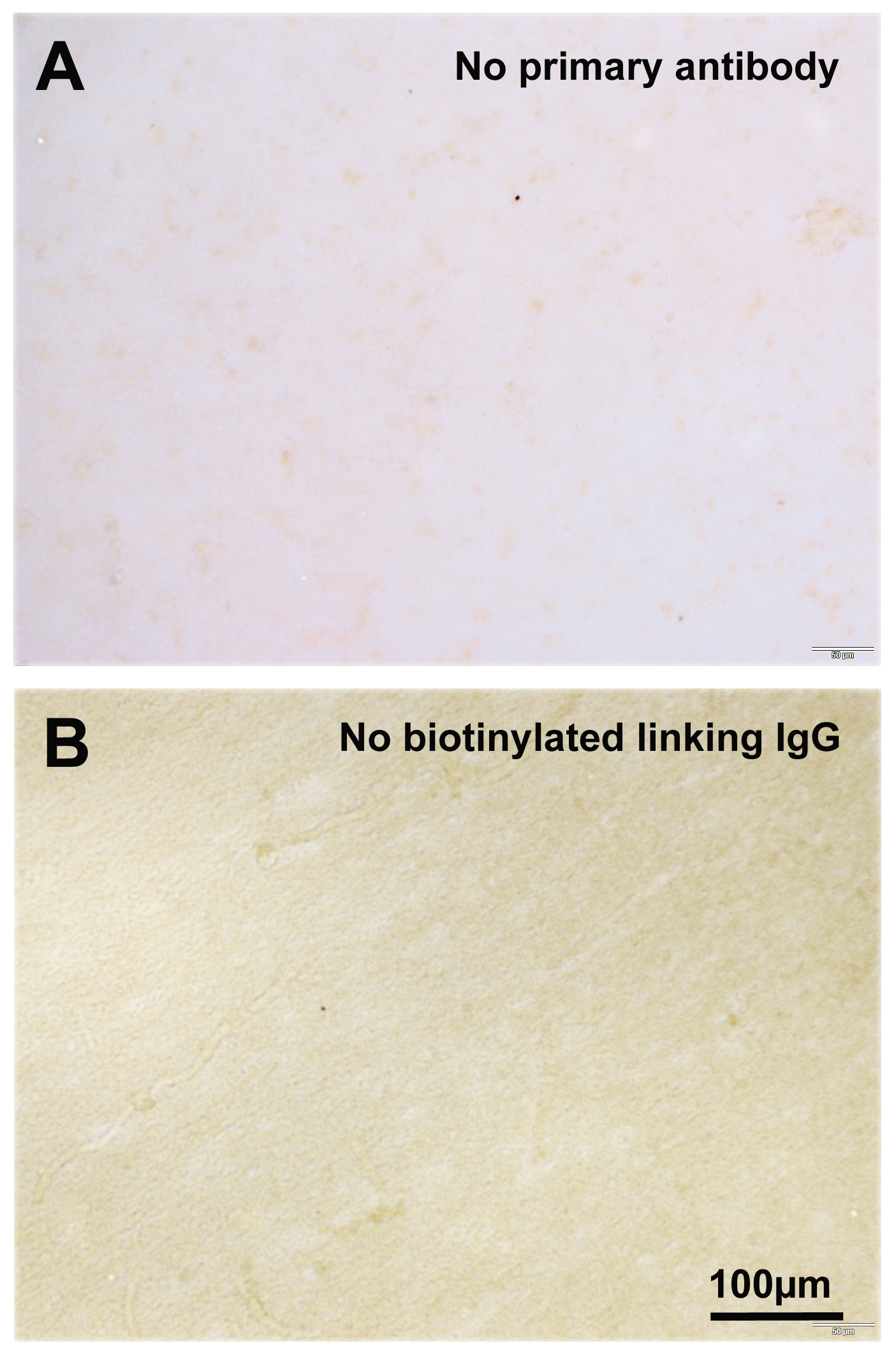

Supplement: SUPPLEMENTARY FIGURE 1 — IHC omission staining. In A, primary antibodies were omitted, the 50μum-thick PFC sections from the 5-year-group were only incubated in biotinylated goat anti-rabbit IgG and Elite ABC solutions, and then were incubated in glucose oxidase-nickel-DAB solutions. In B, the sections were only incubated in a monoclonal rabbit anti-GFAP(ab) antiserum (Abcam, ab68428, 1:2000) while the biotinylated goat anti-rabbit IgG incubation was omitted. Next sections were processed in the following steps as mentioned above. All control sections were treated under the same conditions as those with the primary antisera and the linking IgG. However, following two omission tests, no reliable GFAP(ab)-IR astrocytes were observed in any region of PFC of all brain sections examined. [file Image_1.TIF]

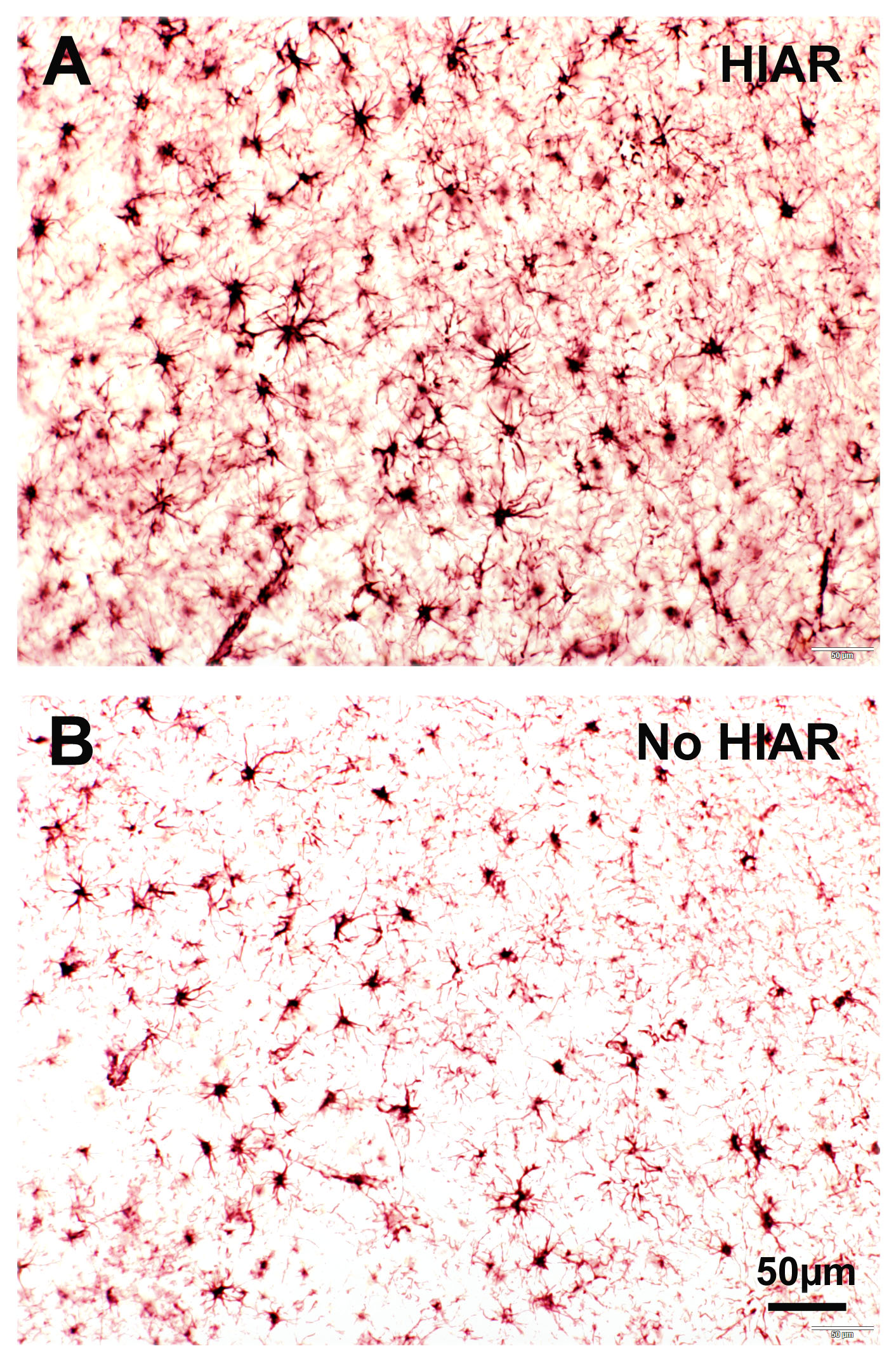

Supplement: SUPPLEMENTARY FIGURE 2 — Expression of GFAP(nb) immunostaining in the CA1 region of hippocampus of a human brain post-fixed for 25 years. Following the treatment of citrate buffer (pH6.0) based heat-induced antigen retrieval (HIAR), abundant strongly stained GFAP(nb)-IR astrocytes were observed in the CA1 of hippocampus (A). However, without HIAR treatment, GFAP(nb)-IR astrocytes were less strong and less abundant in the same region (B) compared to that with HIAR treatment (A). [file Image_2.JPEG]
