# Supplementary material for: Differential effects of prolonged post-fixation on immunohistochemical and histochemical staining for postmortem human brains
Source: Front Neuroanat. 2024 Nov 14;18:1477973. doi: 10.3389/fnana.2024.1477973 (PMC11602276; doi:10.3389/fnana.2024.1477973)
Supplement: Supplementary file 3 [file Data_Sheet_1.PDF]

1. **(ab104224) Anti-NeuN antibody [1B7] - Neuronal Marker**

A monoclonal mouse antibody, supplied by Abcam, raised against RNA binding protein fox-1 homolog 3 (Human), cited in 905 publications.
Immunogen: Recombinant fragment corresponding to Human NeuN aa 1-100 (N terminal). Expressed in and purified from E. coli.Sequence:
MAQPYPPAQYPPPPQNGIPAEYAPPPPHPTQDYSGQTPVPTEHGMTLYTP
AQTHPEQPGSEASTQPIAGTQTVPQTDEAAQTDSQPLHPSDPTEKQQPKR

2. **(ab177487) Anti-NeuN antibody [EPR12763] - Neuronal Marker**

A recombinant monoclonal rabbit antibody, supplied by Abcam, raised against RNA binding protein fox-1 homolog 3 (Human), cited in 1317 publications.
Immunogen: Synthetic peptide. This information is proprietary to Abcam and/or its suppliers.

3. **(NB300-141) GFAP Antibody**

A polyclonal rabbit antibody, supplied by Novus Biologicals (a Bio-Techne brand), raised against Glial fibrillary acidic protein (Human), cited in 93 publications. Applications used include IHC, ICC-IF, WB, IF, and 4 others.
Immunogen: This GFAP Antibody was developed against recombinant full length human GFAP isotype 1 expressed in and purified from E. coli.

4. **(ab68428) Anti-GFAP antibody [EPR1034Y]**

A recombinant monoclonal rabbit antibody, supplied by Abcam, raised against Glial fibrillary acidic protein (Human), cited in 157 publications.
Immunogen: Synthetic peptide. This information is proprietary to Abcam and/or its suppliers.

5. **(ab178847) Anti-Iba1 antibody [EPR16589]**

A recombinant monoclonal rabbit antibody, supplied by Abcam, raised against Allograft inflammatory factor 1 (Human), cited in 430 publications. Applications used include IHC-IF, IHC, WB.
Immunogen: Synthetic peptide. This information is proprietary to Abcam and/or its suppliers.

6. **(ab283346) Anti-Iba1 antibody [EPR16589] - Rat IgG2a**

A rat Recombinant Monoclonal Iba1 antibody. Suitable for IHC-P, ICC/IF, IP, WB, Flow Cyt (Intra) and reacts with Mouse, Rat, Human samples. Cited in 3 publications.
Applications used include IHC-IF, IHC, WB
Immunogen:The exact immunogen used to generate this antibody is proprietary information.
